# Health outcomes and implementation barriers and facilitators of comprehensive geriatric assessment in community settings: a systematic integrative review [PROSPERO registration no.: CRD42021229953]

**DOI:** 10.1186/s12877-022-03024-4

**Published:** 2022-04-29

**Authors:** Grace Sum, Sean Olivia Nicholas, Ze Ling Nai, Yew Yoong Ding, Woan Shin Tan

**Affiliations:** 1 Geriatric Education and Research Institute, Singapore, Singapore; 2 Department of Geriatric Medicine, Institute of Geriatrics and Active Aging, Tan Tock Seng Hospital, Singapore, Singapore; 3 Health Services and Outcomes Research Department, National Healthcare Group, Singapore, Singapore

**Keywords:** Aged, Needs assessment, Functional status, Frailty, Qualitative implementation barriers and facilitators, Systematic integrative review

## Abstract

**Background:**

Comprehensive geriatric assessment (CGA) addresses the bio-psycho-social needs of older adults through multidimensional assessments and management. Synthesising evidence on quantitative health outcomes and implementation barriers and facilitators would inform practice and policy on CGA for community-dwelling older adults.

**Methods:**

We systematically searched four medical and social sciences electronic databases for quantitative, qualitative, and mixed methods studies published from 1 January 2000 to 31 October 2020. Due to heterogeneity of articles, we narratively reviewed the synthesis of evidence on health outcomes and implementation barriers and facilitators.

**Results:**

We screened 14,151 titles and abstracts and 203 full text articles, and included 43 selected articles. Study designs included controlled intervention studies (*n* = 31), pre-post studies without controls (*n* = 4), case-control (*n* = 1), qualitative methods (*n* = 3), and mixed methods (*n* = 4). A majority of articles studied populations aged ≥75 years (*n* = 18, 42%). CGAs were most frequently conducted in the home (*n* = 25, 58%) and primary care settings (*n* = 8, 19%). CGAs were conducted by nurses in most studies (*n* = 22, 51%). There was evidence of improved functional status (5 of 19 RCTs, 2 of 3 pre-post), frailty and fall outcomes (3 of 6 RCTs, 1 of 1 pre-post), mental health outcomes (3 of 6 RCTs, 2 of 2 pre-post), self-rated health (1 of 6 RCTs, 1 of 1 pre-post), and quality of life (4 of 17 RCTs, 3 of 3 pre-post). Barriers to implementation of CGAs involved a lack of partnership alignment and feedback, poor acceptance of preventive work, and challenges faced by providers in operationalising and optimising CGAs. The perceived benefits of CGA that served to facilitate its implementation included the use of highly skilled staff to provide holistic assessments and patient education, and the resultant improvements in care coordination and convenience to the patients, particularly where home-based assessments and management were performed.

**Conclusion:**

There is mixed evidence on the quantitative health outcomes of CGA on community-dwelling older adults. While there is perceived positive value from CGA when carried out by highly skilled staff, barriers such as bringing providers into a partnership, greater acceptance of preventive care, and operational issues could impede its implementation.

**Supplementary Information:**

The online version contains supplementary material available at 10.1186/s12877-022-03024-4.

## Introduction

With a rapidly ageing population globally, there is an unprecedented increase in older adults with complex unmet health and social needs, who require comprehensive care that account for the bio-psycho-social components of health [1]. The comprehensive geriatric assessment (CGA) is an approach developed for this specific purpose [2]. The current literature defines CGA as a multidimensional, multidisciplinary diagnostic and therapeutic process to determine the medical, psychological and functional capabilities of an older person and develop a coordinated and integrated plan for treatment and follow-up [2–4]. This approach to assess older persons recognises the complex interplay of physical illnesses, mental disorders, and the social and home environmental challenges [2, 5]. CGA involves the linkage of medical and social care around medical diagnoses and decision making, and can be nurse-led or led by a doctor trained in geriatric medicine [2, 6].

Existing literature has focused on examining health outcomes of CGA delivered in tertiary care facilities, such as mortality, frailty and functionals status. This is reflected by majority of systematic reviews on CGA that have assessed its impacts on patients receiving care within an inpatient setting, including those admitted to hospital [7, 8], admitted to the emergency department [9, 10] or discharged from the acute hospital [3]. For these populations, CGA was associated with a reduction in the risk of mortality and disability [8]. Importantly, these benefits were only observed in wards specialising in the care of older adults, suggesting that a setting that emphasised geriatric care was crucial [8]. The literature also asserts that older persons who present to the acute hospital tend to have significant functional decline and chronic conditions [11, 12]. CGA for community-dwelling older adults therefore provides the intervention at an earlier stage of the trajectory towards functional disability, chronic disease progression and the need for tertiary care facilities [12]. Acute hospital care has been prioritised for older adults and there is a need to shift this priority towards community-based interventions. This may reduce the burden on hospitals and long-term care facilities, and improve health outcomes [12].

This shift in the need to focus CGA in the community setting is relatively new. Garrard et al. (2020) recently published a systematic review on patient-related outcomes of CGAs conducted in primary care, which only included four studies [4]. The authors revealed that CGA was associated with mixed findings on hospitalisation rates, no difference in functional and survival outcomes, and improved medication adherence [4]. Importantly, the study did not investigate CGA in the setting of patient homes and other community settings [4]. The clinic setting may be relevant for patients who are not receiving care in the hospital. However, home-based CGA is likely relevant for older adults who are frail and experienced functional decline, as well as for those who may not proactively seek medical care [13]. Home-based assessment and care management of older adults have been shown to improve the quality of the evaluation and needs satisfaction of patients [14, 15].

Additionally, community-dwelling older adults encompass persons with varying levels of needs, functional decline and disease progression. It would be useful to examine the impact of CGA conducted on a broader group of older adults for a better representation of patients, and investigate beyond the specific subpopulations that current reviews have focused on such as patients specifically with cancer or frailty [16, 17]. Another knowledge gap is on CGAs conducted by different types of healthcare staff. A systematic review in the midst of being conducted is examining geriatrician-led CGA [18], and even though there is merit in focusing on CGA delivered by specific professionals, it may not be practical to only have geriatricians conducting CGAs. Studies have revealed that trained non-geriatricians could also effectively perform CGAs and lead care programs [19, 20], and utilising a wider range of healthcare staff is likely needed to meet the demands of the rapidly ageing population.

The outcomes of CGA should also be viewed within its implementation context. Another dearth in the literature is the lack of qualitative findings, whereby a few existing systematic reviews have focused on the perspectives of patients and caregivers for CGA conducted in the inpatient setting [21, 22] or with specific chronic conditions [23]. Importantly, even though these reviews reported benefits of CGA, the findings still revealed heterogeneous implementations of CGA and health outcomes [5].

Hence, there are knowledge gaps on CGA conducted in a community setting on older adults that are led by geriatricians and non-geriatricians, as well as qualitative findings of CGA. The objective of the systematic review is to synthesise current evidence on both the quantitative health outcomes and qualitative implementation barriers and facilitators of conducting CGA on community-dwelling older adults.

## Methods

### Search strategy

We systematically searched four medical and social sciences electronic databases (Ovid Medline, Embase, and Cumulative Index of Nursing and Allied Health Literature (CINAHL) and PsycINFO on EBSCO) for articles published from 1 January 2000 to 31 October 2020. We only included articles from the year 2000 so that the reported outcomes are derived from up-to-date health systems and policies. Only articles published in English were included. This language restriction was due to the research team not having access to translators to translate non-English papers. The search was conducted in November 2020 and citations were uploaded to the Covidence online software. Our systematic review protocol is registered with PROSPERO (registration number: CRD42021229953).

The search strategy applied keywords and Medical Subject Headings (MeSH) that were tailored to each database. In summary, key words used for identifying CGA included “geriatric assessment”, “needs assessment”, “geriatric evaluation”, and “geriatric consultation”; key words for identifying older adults included “older adult”, “aged”, “senior”, “elderly”, and “elder”; key words for identifying the CGA setting to in the community included “general practice”, “physicians”, “primary care”, “primary health care”, “community health centres”, “independent living”, “ambulatory care”, “independent living”, aging in place”, “outpatient clinic”, “outpatient care”, “outpatient service”, “day care”, “day rehabilitation”. While we defined CGA as a multidimensional, multidisciplinary diagnostic and therapeutic process to determine the medical, psychological and functional capabilities of an older person and develop a coordinated and integrated plan for treatment and follow-up [2–4], the exact terminology did not have to be used in selected articles and we included studies with two or more assessment domains. The phrase “needs assessment” was used in the search strategy to identify the concept of CGA, the intervention of interest. This is because a key part of the concept of CGA is the comprehensive assessment of needs in different domains of a patient’s life. CGA also involves the development of an individualised care plan. The ability to design this care plan is based on conducting a needs assessment. We applied this less stringent criterion as this is the first systematic review on CGA in the community and is intended to be more inclusive. A recent systematic review on CGA in primary care only had four articles [4]. Furthermore, CGA has been referred to with various terms, such as geriatric assessment, needs assessment [24], or geriatric evaluation and management [25]. Having more than one domain of needs assessment and the development of a care plan to meet those needs [26] were more relevant in the selection of articles for this review. Additional file 1: Appendix A describes the detailed search strategies for each database.

### Inclusion and exclusion criteria

Table 1 shows the detailed inclusion and exclusion criteria.

**Table 1:** Inclusion and exclusion criteria for selected articles

Inclusion		Exclusion
**Study design**		
1.	Primary peer-reviewed articles	Reviews (narrative review, systematic review, meta-analyses, integrative review, umbrella reviews, overviews), conceptual papers, editorials, case studies, position papers, commentaries, protocols. Studies on tool development, validation, concordance, and prediction.
2.	Published from 1 January 2000 to 31 October 2020	Published before 1 January 2000 or after 31 October 2020
3.	In English	Not in English
**Population and setting**		
4.	Older adults aged ≥65 years.	Aged below 65 years. Articles will not be excluded if the study sample is aged younger than 65 years but contains stratified findings for those aged ≥65 years.
5.	Care setting is in the community. i.e., home, transitional care programs at home, primary care, day care centres, day rehabilitation centres, outpatient clinics.	Care setting is not in the community i.e., care setting is not at home, primary care, day care centres, day rehabilitation centres, nor outpatient clinics. Articles are excluded if the care setting is in community hospitals, nursing homes, or other residential facilities. Subjects should not be a warded patient of a community hospital, nursing home or long-term care facility. If CGA is given to community-dwelling adults on an outpatient basis in a setting that happens to be in a community hospital, nursing home or long-term care facility, the article can be included.
	**Intervention: Comprehensive geriatric assessment (CGA)**	
6.	Comprehensive assessment has ≥2 assessment domains. Domains that are assessed include physical health, psychological or mental health, functional status, cognitive status, nutrition, frailty and falls, social health-related (e.g., loneliness), health service utilisation, medication use or polypharmacy, home environment, caregiver burden, financial burden, and self-reported health outcomes like quality of life [1–3]. This list is not exhaustive. The article meets inclusion criteria if the 2 or more domains assessed are sufficiently distinct from each other. **AND** Development of care plan to inform care. The terminology, CGA, can be explicitly or not explicitly used.	Comprehensive assessment has < 2 assessment domains. **OR** No development of care plan to inform care.
7.	CGA is not conducted for the purpose of addressing a single specific health condition or health issue.	CGA is conducted to address a specific health condition or health issue. For example, articles are excluded if the aim of the CGA intervention is to only address falls, mental health conditions, cancer, neurological conditions, pre-operative issues, self-neglect, etc.
**Comparator**		
8.	There is no inclusion criteria for comparator. Articles with and without a comparator group (i.e. control group) that does not have the CGA intervention can be included.	NA
**Outcome**		
9.	Quantitative health outcomes (Health outcomes refer to indicators to changes in health status) **AND/OR** Qualitative implementation barriers and facilitators of CGA	No quantitative health outcome **AND** No qualitative implementation barriers and facilitators of CGA
	For clarity, the following will be included: ▪ Quantitative articles on only health outcomes ▪ Qualitative articles on only implementation barriers and facilitators of CGA ▪ Mixed methods articles on only quantitative health outcomes ▪ Mixed methods articles on only implementation barriers and facilitators of CGA ▪ Mixed methods articles on both quantitative health outcomes and implementation barriers and facilitators of CGA	

*CGA* Comprehensive geriatric assessment

### Study selection

There were four reviewers involved in study selection: Sum G, Nicholas SO, Nai ZL, and Tan WS. Titles and abstracts were independently reviewed by any two of the four reviewers. Disagreement was resolved by a third vote from any of the remaining two reviewers. Sum G, Nicholas SO, and Nai ZL screened full texts articles for eligibility, and each article was independently reviewed by two of the three reviewers. Disagreements were resolved through consultation with Tan WS.

### Data extraction

Each included article was assigned to either Sum G, Nicholas SO or Nai ZL for both data extraction and quality evaluation. We extracted data including reference information (author, year, country), study design and population, length of follow-up, setting of CGA, CGA domains and personnel who conducted the CGA, usual care to controls, objective of intervention, health outcomes, other quantitative outcomes, and qualitative implementation barriers and facilitators.

### Quality evaluation

We assessed the quality of the included controlled intervention studies, pre-post studies without controls, and case-control studies were conducted using the National Institute of Health (NIH) quality assessment tools relevant to each type of study design [27]. Assessment domains included accurate descriptions of study designs, methodology (e.g. whether treatment allocation was concealed for controlled intervention studies, whether eligibility criteria were pre-specified for the study population for pre-post studies, whether controls were selected from the same population that gave rise to cases for case-control studies), outcome measures (e.g. whether the outcome measures were pre-specified, valid and reliable), and statistical analyses (e.g. did authors use an intention-to-treat analysis for controlled intervention studies, were statistical tests done to provide *P*-values for pre-post changes, were key confounders measured and adjusted statistically). Options included yes (1 point), no (0 points), cannot determine, not reported, and not applicable (0 points each) [27]. Controlled intervention studies and pre-post studies were categorised into good (10 to 14 points), fair (7 to 9 points) and poor (0 to 6 points) quality; and case-control articles were categorised into good (9 to 13 points), fair (6 to 8 points) and poor (0 to 5 points) [27].

Quality assessment of qualitative articles was conducted using the 10-item Critical Appraisal Skills Program (CASP) for qualitative research [28]. Domains included the clarity of research aims, appropriateness of using qualitative methods and research design, recruitment strategy, data collection, ethics, and data analysis [28]. Articles were categorised into good (7 to 9 points), fair (5 to 6 points) and poor (0 to 4 points) quality. Mixed methods articles were assessed using the 5-item Mixed Methods Appraisal Tool (MMAT) [29]. Domains pertained to the rationale for using mixed methods, whether the quantitative and qualitative components were effectively integrated and interpreted, and quality of each of the different components. Articles were categorised into good (4 to 5 points), fair (3 points) and poor (0 to 2 points) quality. Additional file 2: Appendix B shows the details of the quality assessment questions [29].

### Data analysis

The selected articles were heterogeneous in study design, sample population, settings of CGA, and assessment of health outcome measures. Due to this heterogeneity, we did not conduct a meta-analysis of the effect sizes. Instead, we narratively synthesised the evidence on health outcomes and presented qualitative findings based on themes of implementation barriers and facilitators. Quantitative findings were presented by categories of health outcomes, including functional status, frailty and falls, mental health, self-rated health, cognition, chronic conditions, medicine use, nutritional status, quality of life, and mortality. Qualitative findings from individual studies were extracted into a database and subsequently categorised based on whether it described barriers and facilitators of CGA implementation. We summarised the main ideas and conclusions identified by the studies’ authors and developed themes inductively by relating similar concepts from one study to another [30]. Acknowledging the subjective perspectives of stakeholders such as healthcare professionals and patients, the results were initially collated separately. However, due to significant overlaps in both perspectives, our results did not differentiate across the two groups.

## Results

### Selected articles

**Fig. 1:**
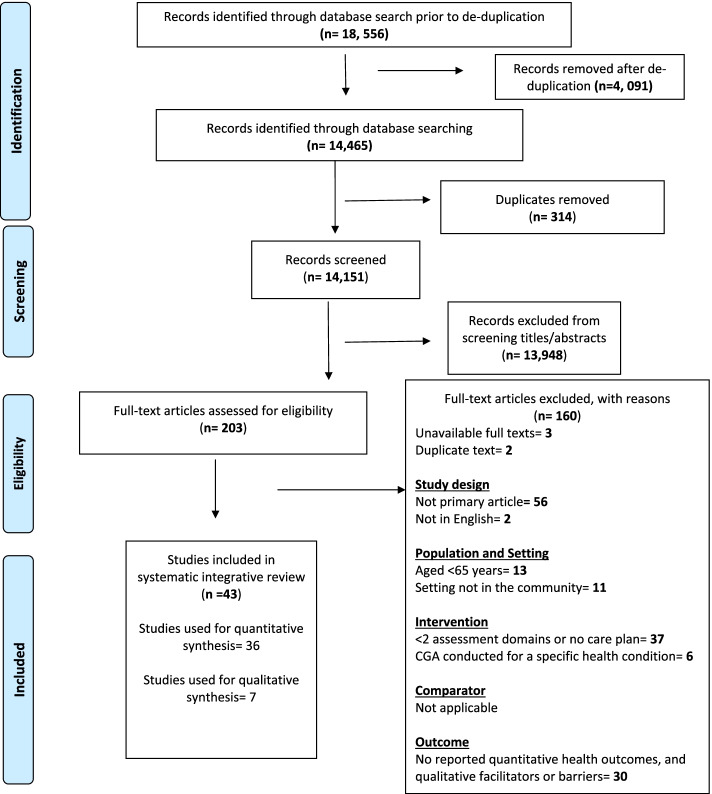
Preferred Reporting Items for Systematic Reviews and Meta-Analyses (PRISMA) flowchart

14,465 records were identified from database searches. There were 14,151 records after removing duplicated articles, which were screened based on titles and abstracts. We assessed 203 full text articles for eligibility and 43 primary articles were included in this systematic review. Figure 1 shows the Preferred Reporting Items for Systematic Reviews and Meta-Analyses (PRISMA) flowchart.

### Characteristics of selected articles

The characteristics of the 43 studies are summarised in Table 2. Study designs included controlled intervention studies (*n* = 31, 72.1%) which included RCTs (*n* = 30) and controlled pre-post (*n* = 1), pre-post studies without controls (*n* = 4, 9.3%), case-controls (*n* = 1, 2.3%), qualitative methods (*n* = 3, 7.0%), and mixed methods (*n* = 4, 9.3%). Studies were conducted in Europe (53.5%), United States (US) (20.9%), Canada (7.0%), Australia (4.7%), New Zealand (4.7%), Hong Kong (2.3%), South Korea (2.3%), and Taiwan (2.3%). Most articles were published from 2016 to 2020 (*n* = 16, 37%). Majority studied populations aged ≥75 years (*n* = 18, 42%) and ≥ 65 years (*n* = 11, 25%). The most common setting for conducting CGA was at home (*n* = 25, 58%), followed by primary care (*n* = 8, 19%), and in a secondary or tertiary care setting on an outpatient basis (*n* = 5, 12%). CGAs were conducted by nurses in most studies (*n* = 22, 51%), followed by geriatrician and/ nurse and/or social worker (*n* = 7, 17%), and a multidisciplinary team (*n* = 6, 14%).

**Table 2:** Characteristics of selected articles (*n* = 43)

Characteristic	Controlled intervention studies (***n*** = 31)		Pre-post without controls (***n*** = 4)	Case control (***n*** = 1)	Qualitative (***n*** = 3)	Mixed methods (***n*** = 4)	Total (***n*** = 43)
	Randomised controlled trials (***n*** = 30)	Controlled pre-post study (***n*** = 1)					
**Publication year**							
2000–2005	8				1	2	11 (26%)
2006–2010	7		2	1			10 (23%)
2011–2015	4		1			1	6 (14%)
2016–2020	11	1	1		2	1	16 (37%)
**Country**							
United States	6		1	1		1	9 (21%)
Canada	3						3 (7%)
Denmark	1						1 (2%)
Finland	1						1 (2%)
Italy			1				1 (2%)
Netherlands	7				1	1	9 (21%)
Norway	1						1 (2%)
Spain	1						1 (2%)
Sweden	4					1	4 (9%)
Switzerland	2						2 (5%)
Switzerland and Netherlands			1				1 (2%)
United Kingdom	1					1	2 (5%)
Australia	1				1		2 (5%)
New Zealand		1			1		2 (5%)
Hong Kong	1						1 (2%)
South Korea			1				1 (2%)
Taiwan	1		1				2 (5%)
**Population age (years)**							
≥65	8		3				11 (25%)
> 65				1	1		2 (5%)
≥70	4						4 (9%)
> 70	2						2 (5%)
≥75	12	1	1		2	2	18 (42%)
> 75						2	2 (5%)
≥80	3						3 (7%)
70 to 84	1						1 (2%)
**Where CGA is conducted**							
Home	18	1	1		2	3	25 (58%)
Primary care setting: General practice, elderly medical centre	5		1	1		1	8 (19%)
Home and general practice	1						1 (2%)
Home and phone	2						2 (5%)
Secondary or Tertiary care setting: Outpatient geriatric clinic, outpatient geriatric unit, outpatient geriatric medical centre	3		2				5 (12%)
Geriatric clinic and phone	1						1 (2%)
Location not specified (but care setting confirmed to be in the community)					1		1 (2%)
**CGA conducted by**							
Nurse	16	1	2		1	2	22 (51%)
Geriatrician	1			1			2 (5%)
Geriatrician, nurse	2					1	3 (7%)
Geriatrician, nurse, social worker	1		1				2 (5%)
General practitioner, nurse	1				1		2 (5%)
Nurse, occupational therapist	1						1 (2%)
Physiotherapist, social worker			1				1 (2%)
Nurse, physiotherapist, social worker	1						1 (2%)
Nurse, social worker, pharmacist	2						2 (5%)
Multidisciplinary team	4				1	1	6 (14%)
Trained interviewers	1						1 (2%)

### Quality of selected articles

Articles were categorised as good (*n* = 23, 53.5%), fair (*n* = 16, 37.2%), and poor (*n* = 4, 9.3%) quality. Controlled intervention studies were categorised as good (*n* = 19, 61.3%), fair (*n* = 9, 29.0%), and poor (*n* = 3, 9.7%) quality. All pre-post studies without controls (*n* = 4, 100%) were fair quality. The case-control study (*n* = 1, 100%) was categorised as good quality. Qualitative articles were good (*n* = 2, 66.7%) and fair (*n* = 1, 33%). Mixed methods articles were good (*n* = 1, 25.0%), fair (*n* = 2, 50.0%), and poor (*n* = 1, 25.0%). Additional file 2: Appendix C shows the scoring of the quality evaluation of articles.

### Quantitative health outcomes

Table 3 summarises the data extracted from the 36 articles that reported quantitative health outcomes from the CGA intervention (31 controlled intervention studies, four pre-post studies without controls, one case-control), including reference information, study design and setting, where CGA was conducted, personnel who conducted CGA, components of CGA, and health outcomes. Additional file 3: Appendix D shows the detailed data extraction of these studies.

**Table 3:** Summary of quantitative health outcomes (*n* = 37)

Reference, Country	Design, Length of follow up, Setting	Comprehensive geriatric assessment (CGA)		Quantitative health outcomes
		Conducted at, conducted by	Components	
Avlund et al. 2002 [31] Denmark Quality: Poor	RCT, 3 months 32 persons aged > 70 years at the medical wards.	At home Conducted by: General practitioner, home nurse, home helper, physiotherapist, and/or occupational therapist.	Health and medical problems.	**Functional status** (mean Barthel Index): ↑
Ballabio et al. 2008 [32] Italy Quality: Fair	Pre-post, 3 months 222 persons aged ≥75 years who were discharged from the emergency department.	Outpatient geriatric unit. Conducted by: Geriatrician, nurse and social worker.	Physical status, functional status, cognitive status, depression, cognitive dysfunction, caregiver stress, perceived QoL.	**Functional status** (ADLs, IADLS): > = < **Quality of life** (EuroQoL Analogic section): ↑ **Mental health** (Cornell scale and GDS): ↑ **Cognition:** All patients: > = < Among those with cognitive dysfunction): ↑ **Chronic conditions** (cumulative illness rating scale): > = < **Nutritional status** (MNAS): ↑
Bleijenberg et al. 2017 [33] Switzerland and Netherlands Quality: Fair	Pooled analysis of 2 RCTs, 9 months and 12 months. 461 persons aged ≥80 years, whereby 230 from UPROFIT had multimorbidity, polypharmacy and care gap in primary care of ≥3 years, and 231 from HCP were from healthcare organisations, local hospitals and social services.	At home and primary care Conducted by: Advance practice registered nurses.	Clinical assessment of health and family situation, symptoms of illness, frailty and falls, urinary incontinence, cognition, loneliness.	**Functional status:** ↑
Blom et al. 2016 [34] Netherlands	RCT, 1 year 59 practices with 7278 participants aged ≥75 years	General practice Conducted by: General practitioner or practice nurse.	Functional, somatic (health and illness), mental, and social Each domain contained 4–9 questions.	**Functional status** (BADLs and IADLs via GARS): > = < **Quality of life** (Cantril’s ladder): > = < **Mental health** (GDS-15, Loneliness scale of DJG): > = <
Boult et al. 2001 [35] United States Quality: Good	RCT, 18 months 568 persons aged ≥70 years at a high risk of poor functional ability and high use of health services.	At an outpatient geriatric evaluation and management clinic (ambulatory clinic) in a community hospital. Conducted by: General nurse practitioner, geriatrician, and nurse.	Medical conditions, psychosocial status, functional ability, cognitive status, nutritional risk, use of alcohol, social network, gait and balance, environmental safety, medications, advance directives, hearing, vision.	**Functional status:** ↑ **Mental health** (GDS): ↑ **Mortality rate:** > = <
Boult et al. 2013 [36] United States Quality: Good	RCT, 32 months 904 persons aged ≥65 years t high risk of using health services heavily during the following year, as estimated by the claims based hierarchical condition category (HCC) predictive model.	At home Conducted by: registered nurses with at least 3 years of clinical experience and who took a Guided Care preparatory course.	Not specified, but comprehensive assessments were conducted with individualised action plans designed.	**Quality of life** (physical and mental scores)**:** > = < **Mortality rate:** > = < **Self-rated health status:** > = <
Bouman et al. 2008 [37] Netherlands Quality: Good	RCT, 18 months 293 persons aged 70 to 84 years who lives at home and has poor health status.	At home Conducted by: Home nurses (auxiliary community nurses) under the supervision of public health nurses (community nurses).	Health problems and risks.	**Functional status** (ADLs): > = < **Quality of life:** > = < **Self-rated health status**: > = <
Burns et al. 2000 [38] United States Quality: Fair	RCT, 2 years 128 persons aged ≥65 years with ADL deficits, chronic conditions, acute care hospitalisations in previous year, and on scheduled prescriptions.	Geriatric primary care Conducted by: Interdisciplinary care team.	Health status including mortality, global health perception, clinic visits and hospitalisations, functional status, global social activity, quality of life, life satisfaction, and cognition.	**Functional status** (IADL impairments, ADL scores): > = < **Mortality rate:** > = < **Mental health** (CES-D, well-being, life satisfaction): ↑ **Health status** (health perception**):** ↑ **Cognition** (MMSE): ↑
Byles et al. 2004 [39] Australia Quality: Good	RCT, 3 years 1082 veterans or war widows receiving full entitlements from the Australian Department of Veterans’ Affairs, and aged ≥70 years.	At home Conducted by: Nurses, social workers, psychologists, physiotherapists, and/or occupational therapists.	Use of hearing aids, vision, dental care and dentures, vaccinations, prescribed and over-the-counter medications, hypertension management, diabetes management, smoking status and desire to quit, BMI, problems with feet, problems with leaking urine, self-rated health, difficulty sleeping, use of community services, ANISIC, Medical Outcomes Study physical function scale (items to assess mobility), brief MMSE, Duke Social Support Index, Modified GDS.	**Quality of life** (physical and mental component summary scores): ↑ **Mortality rate:** > = <
Chi et al. 2006 [40] Hong Kong Quality: Poor	RCT, 12 months 925 older Chinese adults aged ≥65 years who attend the elderly health centres of the department of Health, Hong Kong special Administrative Region.	At the elderly health centre Conducted by: Trained interviewers.	General functioning, cognitive function, social support, physical functioning, physical illnesses, living environment, formal service utilisation, medication.	**Functional status** (ADLs, IADLs, stamina): > = < **Mental health** (mood, behaviour): ↑ **Cognition:** > = <. **Chronic conditions:** Pain symptoms, pressure ulcer, bladder incontinence: > = < Bowel incontinence: **↓**
Cohen et al. 2002 [41] United States Quality: Good	RCT, 1 year 1388 persons aged ≥65 years who were hospitalised on a medical or surgical ward, had length of stay of at least two days, and frailty.	Outpatient geriatric evaluation and management Conducted by: Geriatrician, social worker, and nurse.	Medical history and physical examination, functional, cognitive, affective, and nutritional status, caregiver’s capabilities, patient’s social situation, and geriatric syndromes such as incontinence or falls.	**Functional status** (BADLs, IADLs, physical performance): > = < **Quality of life** SF-36 scores for physical functioning, physical limitations, emotional limitations, and social activity, bodily pain: > = < SF-36 scores for energy, general health and mental health: ↑ **Mortality rate**: > = <
Eckerbald et al. 2016 Sweden Quality: Fair	RCT, 24 months 242 persons aged ≥75 years with 3 or more concomitant medical diagnoses and 3 or more hospitalisations during the preceding year.	At home Conducted by: Trained registered nurses or a registered occupational therapist.	Medical, psychological, functional.	**Chronic conditions** (MSAS): > = <
Ekdahl et al. 2015 [42] Sweden Quality: Good	RCT, 24 months 382 persons (208 intervention, 174 controls) aged ≥75 years who received inpatient hospital care 3 or more times in the previous 12 months and had 3 or more concomitant medical diagnoses.	At home Conducted by: Registered nurse and registered occupational therapist.	Hearing and vision problems, independence in ADLs, cognition, sense of security in care, health-related quality of life	**Quality of life** (HR-QoL): > = < **Mortality rate:** > = <
Ekdahl et al. 2016 [43] Sweden Quality: Good	RCT, 36 months 382 persons (208 intervention, 174 controls) aged ≥75 years who received inpatient hospital care 3 or more times in the previous 12 months and had 3 or more concomitant medical diagnoses.	At home Conducted by: Registered nurse and registered occupational therapist.	Hearing and vision problems, independence in ADLs, cognition, sense of security in care, health-related quality of life	**Mortality rate:** ↑ (= improved = decreased mortality)
Faul et al. 2009 [44] United States Quality: Fair	Pre-post, 12 weeks 73 persons aged ≥65 years with chronic conditions and no ongoing home health care.	At home Conducted by: Physical therapist, physical therapist student, social worker student.	Cognition, functional status, physical mobility, mental health, physical home environment, chronic diseases, self-management, self-rated health.	**Functional status** (function and physical mobility): ↑ **Falls** (physical home environment and falls hazards) ↑ **Mental health** (GDS): ↑ **Self-rated health status**: ↑ **Chronic conditions** (self-efficacy for chronic disease management) > =<
Fenton et al. 2006 [45] United States Quality: Good	Case control, 20 months 583 persons (146 cases, 437 controls) aged ≥65 years who attended the 2 physician practices in the study and enrolled into the health plan from 2 years before their index visit with the geriatrician until either death or the end of the study.	Primary care practice Conducted by: Fellowship-trained geriatrician.	(1) standardized assessment of psychosocial, cognitive, and physical function and physical activity (2); screening for pain, depression, dementia, urinary incontinence, fall risk, and substance abuse (3); review for use of medications with frequent adverse side effects in elderly patients; and (4) focused physical examination.	**Mortality rate:** > = < **Medications** (rate of high-risk prescriptions): > = <
Fletcher et al. 2004 [19] United Kingdom Quality: Good	RCT, 3 years 8797 persons aged ≥75 years from the general population.	At home Conducted by: Nurse.	Cognition, mental health, functional, physiological, social	**Quality of life** (mobility, social interaction and morale compared): ↑ **Mortality rate:** > = <
Godwin et al. 2016 [13] Canada Quality: Poor	RCT, 12 months 143 persons aged ≥80 years functioning well cognitively and living independently in the community.	At home Conducted by: Primary Care Nurse Specialist	ADLs and IADL, symptomatology, medication usage, compliance and knowledge by medication review, safety issues, including risk of falls, use of stoves and other potentially dangerous appliance, general home and personal hygiene and maintenance, understanding of their medical/health conditions to determine their need for education, and need for community services.	**Quality of life** (SF-36, CASP-19): > = < **Chronic disease** (symptomology using the Comorbidity Symptom Scale): > = <
Hebert et al. 2001 Canada Quality: Good	RCT, 1 year 494 persons on the Quebec Home Insurance Plan aged ≥75 years	At home Conducted by: Trained nurse.	Medication, cognitive function, depression, balance or risk of falling, orthostatic hypotension, environmental risks, social support, nutrition, arterial hypertension, vision, hearing, incontinence.	**Functional status** (SMAF, relative risk of functional decline): > = < **Mental health** (DGWBS on anxiety, depression, positive well-being, self-control, vitality, and general health): > = <
Hoogendijk et al. 2016 [46] Netherlands Quality: Good	RCT, 24 months 1147 patients across 35 primary care practices, aged ≥65 years, and had a PRISMA-7 score of 3 or more.	At home Conducted by: Practice care nurse	Identification of care needs and health risks, including preventive health, Cardio-respiratory conditions, health promotion, depression and anxiety, urinary incontinence, pain, social functioning, falls, tobacco and alcohol use, medication management.	**Functional status** (ADL, IADL): > = < **Quality of life** (SF-12, EQ-5D): > = < **Mental health** (psychological well-being via 5-item RAND-36 mental health subscale): ↑ **Self-rated health status:** > = <
Imhof et al. 2012 [47] Switzerland Quality: Fair	RCT, 9 months 413 persons aged ≥80 years who are german-speaking.	At home Conducted by: Advanced practice nurse.	Demographic variables, living situation, family network, and health status (mobility and falls, pain, vision and hearing ability, sleep pattern, bladder control, nutritional status, substance use, cognition, and use of medications and aides for mobility). Clinical tests were included for vision (Amsler-Gitter Test), gait, balance, and strength, tandem stand, timed five-chair-rise test, and screening for malnutrition (Mini Nutritional Assessment), and depression (GDS-4).	**Quality of life:** > = < **Frailty/falls:** ↑ (lower relative risk of falls and consequences of falls) **Acute adverse events:** ↑ (lower relative risk of acute events, defined as acute health symptoms that required action)
Kang et al. 2020 [48] South Korea Quality: Fair	Pre-post, mean of 5.1 months 362 persons aged ≥65 years who regularly visited primary medical institutions at the regions where study was conducted.	Outpatient medical centre and public health centre Conducted by: Trained nurses	Comorbidity, physical function, cognitive function, quality of life, drugs, and nutrition.	**Functional status** (physical function, gait speed, grip strength): ↑ **Quality of life** (ED-5D): ↓ **Polypharmacy:** ↑ (decreased proportion with polypharmacy). **Nutrition (MNA):** ↑ (decreased risk of malnutrition or being malnourished).
King et al. 2018 [49] New Zealand Quality: Fair	Controlled before-after study, 1 year before and after intervention 1400 persons aged ≥75 years enrolled in one of the primary healthcare practices that will implement the new care model.	At home Conducted by: Specialist Gerontology Nurse.	Body systems (respiratory, cardiac, neurological, gastrointestinal, musculoskeletal and bladder/bowel function), pain, medications, potential social issues, functional ability, cognitive impairment, depression.	**Mortality rate:** > = <
Li et al. 2010 [50] Taiwan Quality: Fair	RCT, 6 months 310 persons aged ≥65 years living in neighbourhoods within 15 min walking distance from the community hospital.	Community hospital on an outpatient basis Conducted by: Nurses.	Geriatric syndromes (falls, incontinence, polypharmacy, sleep disturbance, nutrition, pain); cognition; depression; nutrition; functional (visual acuity); physical; orthostatic hypotension screening.	**Functional status** (Barthel Index): > = < **Frailty** (likelihood to have a better outcome and likelihood to deteriorate in frailty status measured with FFC): > = <
Liimatta et al. 2019 [51] Finland Quality: Fair	RCT, 2 years 422 persons aged ≥75 years not receiving home help or nursing services.	At home. Conducted by: Nurse, physiotherapist, social worker.	Functioning, Mental Capability, health status, health and social services present, mobility, strength, ADLs, IADLs, financial and other social service needs.	**Quality of life** (15-dimensioanl assessment scale)**:** ↑ **Mortality rate:** > = <
Lin et al. 2012 [52] Taiwan Quality: Fair	Pre-post, 12 months Total of 140 persons aged ≥80 years with any health conditions, and aged ≥65 years with multiple complex care needs, or more than 3 co-morbid chronic diseases, or with geriatric syndrome.	Outpatient geriatric evaluation and management service in Taipei Veterans General, a tertiary medical centre. Conducted by: Research nurses	Physical Function, IADLs, cognitive function, mood status, delirium, falls, incontinence nutritional status, QoL, social care resource.	**Quality of life:** ↑ (QoL gained from the service model was estimated to be 4.1 QALY) **Polypharmacy:** ↑ (reduction in no. long-term medications)
Mazya et al. 2019 [53] Sweden Quality: Good	RCT, 24 months 360 persons aged ≥75 years with 3 or more chronic conditions and 3 or more inpatient admissions the past 12 months.	At home and via phone Conducted by: Nurse and social worker (home), pharmacist (phone).	Medical, functional, psychological, cognitive, social.	**Mortality rate:** > = < **Frailty:** ↑ (intervention group had lower proportion of frail patients, higher proportion of pre-frail patients)
Monteserin et al. 2010 [54] Spain Quality: Good	RCT, 18 months 620 persons aged ≥75 years who has access to primary care health centre.	Primary care health centre Conducted by: Nurse.	Socio-demographics, perceived health status, sensory evaluation (sight and hearing), falls, urinary incontinence, prescribed medications, comorbidity, functional status, IADL, neuropsychological status, cognitive status, nutritional status and social support.	**Mortality rate:** > = < **Frailty**: ↑ (intervention group had proportion of frail patients who went from not at risk for frailty to at risk for frailty, greater proportion who reserved frailty status)
Ploeg et al. 2010 [55] Canada Quality: Good	RCT, 12 months 719 persons aged ≥75 years at risk of functional decline.	At home Conducted by: Nurse.	QoL, health status, costs of health and social services, functional status, self-rated health.	**Functional status** (ADLs): > = < **Quality of life** (QALY): > = < **Self-rated health status:** > = <
Romskaug et al. 2020 [56] Norway Quality: Good	RCT, 24 weeks 158 persons aged ≥70 years who used at least 7 systemic medications taken regularly, and had their medications administered by the home nursing service.	Primary care practice Conducted by: Physician trained in geriatric medicine, supervised by a senior consultant.	Medical history, systematic screening for current problems, clinical examination of the patient, relevant supplementary test, and detailed review of each medication in use, with emphasis on indication, dosage, possible adverse effects, and interactions.	**Functional status** (functional independence measure): > = < **Quality of life:** > = < **Falls** (number of falls): > = < **Mortality rate:** > = < **Mental health** (relative stress, the number of days the patient spent in his or her own home during follow-up): > = < **Chronic conditions:** Orthostatic blood pressure, weight): > = < **Medication appropriateness** (assessed by the Medication Appropriateness Index and the Assessment of Underutilization): ↑
Rubenstein et al. 2007 [57] United States Quality: Good	RCT, 3 years 532 persons aged ≥65 years who had at least one clinic visit at the ambulatory centre in the previous 18 months and deemed high risk (impaired in 4 or more of 10 Geriatric Postal Screening Survey questions).	Over the phone and at a geriatric assessment clinic Conducted by: Physician assistant case manager (phone), and geriatric medicine faculty, physician assistant, and internal medicine house staff (at clinic).	Physical health, functional status, mental health, social and environmental status.	**Functional status** (ADLs, IADLs): > = < **Falls** (prevalence or severity of falls): > = < **Chronic conditions** (prevalence or severity of urinary incontinence): > = < **Mental health:** > = < **Self-rated health status:** > = <
Stuck et al. 2000 [58] Switzerland Quality: Good	RCT, 3 years 791 persons aged ≥75 years in the health insurance list of community-dwelling residents in three zip code areas of Bern, and categorised as high-risk and low-risk for nursing home admission.	At home Conducted by: 3 certified registered nurses with an additional degree in public health nursing based on an 8-month postgraduate course (Nurse A, B, C)	Medical history, physical examination, haematocrit and glucose levels in blood, hearing, vision, nutritional status, oral health, appropriateness of medication use, safety in the home, ease of access to external environment, social support.	**Functional status**: Among low-risk subjects: ↑ Among high-risk subjects: > = < Among low-risk subjects visited by nurse A and nurse B: ↑
Suijker et al. 2016 [20] Netherlands Quality: Good	RCT, 24 months 2283 persons aged ≥70 years with complex care needs.	At home Conducted by: Community-care registered nurse.	Somatic, psychological, functional, and social.	**Functional status** (Katz-ADL index scores): > = < **Quality of life** (HR-QoL, emotional well-being, self-perceived QoL): > = < **Mortality:** > = < **Falls** (number of falls): > = <
Suijker et al. 2017 [59] Netherlands Quality: Good	RCT, 12 months 2283 persons aged ≥70 years with complex care needs.	At home Conducted by: Community-care registered nurse.	Somatic, psychological, functional, and social.	**Functional status** (Katz-ADL index scores): > = < **Quality of life** (QALY): > = <
van Hout et al. 2010 [60] Netherlands Quality: Good	RCT, 18 months 424 persons aged ≥75 years with frailty.	At home Conducted by: Trained community nurse.	Health risks and care needs using the Resident Assessment Instrument–Home Care version (RAI-HC).	**Functional status** (ADLs, IADLs): > = < **Mortality rate:** > = <
van Leeuwen et al. 2015 [61] Netherlands Quality: Fair	RCT, 24 months 1147 frail older adults aged ≥65 years with PRISMA-7 scores of 3 or more.	At home Conducted by: Registered nurses with experience in geriatric nursing.	Health and care needs identified from the web-based Community Health Assessment version 9.1 of the Resident Assessment Instrument.	**Functional status** (ADLs, IADLs: > = < **Quality of life** (SF-12 physical and mental scales)**:** > = <

1. Ballabio C, Bergamaschini L, Mauri S, et al. A comprehensive evaluation of elderly people discharged from an Emergency Department. *Intern Emerg Med* 2008; **3(**3): 245–9

2. Boult C, Boult LB, Morishita L, Dowd B, Kane RL, Urdangarin CF. A randomized clinical trial of outpatient geriatric evaluation and management. *J Am Geriatr Soc* 2001; **49(**4): 351–9

3. Faul AC, Yankeelov PA, Rowan NL, et al. Impact of geriatric assessment and self-management support on community-dwelling older adults with chronic illnesses. *J Gerontol Soc Work* 2009; **52(3)**: 230–49

↑: Better; ↓: Worse; > = <: No statistically significant difference

*ANSIC* Australian Nutrition Screening Initiative Checklist, *ADL* Activities of Daily Living, *BADL* Basic Activities of Daily Living, *BI* Barthel Index, *CES-D* Centre for Epidemiological Studies- Depression, *CGA* Comprehensive Geriatric Assessment, *DGWBS* Depuy’s General Well-being Schedule, *FFC* Fried Frailty Criteria, *GARS* Groningen Activity Restriction Scale, *GDS* Geriatric Depression Scale, *HR-QoL* Health-Related Quality of Life, *IADL* Instrumental Activities of Daily Living, *MMSE* Mini Mental State Examination, *MNAS* Mini-Nutritional Assessment Scale, *MSAS* Memorial Symptom Assessment Scale, *PRISMA* Program of Research to Integrate the Services for the Maintenance of Autonomy, *SMAF* Functional Autonomy Measurement System, *QoL* Quality of Life

#### Functional status outcomes

Nineteen RCTs [6, 20, 31, 33–35, 37, 38, 40, 41, 46, 50, 55–61] and three pre-post studies without controls [32, 44, 48] examined functional status as an outcome of CGA. All included RCTs assessed functional status based on independence in the performance of a pre-defined list of Activities of Daily Living (ADL) and Instrumental Activities of Daily Living (IADL) in the intervention group compared to controls. Fourteen RCTs (73.7%) found no difference in the sum of counts of dependence in ADLs/IADLs in the intervention group, compared to controls [6, 20, 34, 37, 40, 41, 46, 50, 55–57, 59–61], while the remaining five RCTs (26.3%) found improvement in the sum of counts of dependence in ADLs/IADLs in the intervention group, compared to controls [31, 33, 35, 38, 58]. Settings were heterogeneous, whereby CGA was conducted at patients’ homes (*n* = 2) [31, 58], at the primary care clinic (*n* = 1) [38], outpatient geriatric clinic (*n* = 1) [35], and both at home and primary care (*n* = 1) [33]. CGA was conducted by nurses (*n* = 2) [33, 58], jointly by a nurse and geriatrician (*n* = 1) [35], and a multidisciplinary team (*n* = 2) [31, 38].

Two of the three pre-post studies reported significantly improved functional ability via measures of mobility, balance, gait speed and strength [48], and lower extremity muscle strength related to ambulation and stair climbing [44]. Kang et al. (2020) was a fair quality study conducted in South Korea, which examined CGAs conducted by trained nurses at an outpatient medical centre and public health centre for older adults [48]. The study reported a significant reduction in the mean time of the Timed Up and Go (TUG) test and an increase in the mean gait speed for all participants, and man increase in the mean grip strength of female participants [48]. Faul et al. (2009) was a fair quality study conducted in the US [44]. It investigated the outcomes of CGAs conducted by a physical therapist, a physical therapists student, and social worker student at the homes of patients with chronic conditions [44]. The authors revealed a significant improvement in the Timed Sit to Stand test, however, there were no differences in limitations of dynamic balance and agility assessed via the Functional Reach test and TUG, and the sum of counts of dependence in IADLs [44].

#### Frailty status and incidence and severity of falls

Six RCTs [47, 50, 53, 54, 56, 57] and one pre-post study [44] examined frailty and falls. Only half of the RCTs reported no impact of CGA on these outcomes in the intervention group, compared to controls. The articles measured frailty using the Fried Frailty Criteria (FFC) [50], number of falls [56, 57] and severity of falls [57]. The remaining three RCTs assessed the risk of falls and the prevalence of adverse fall consequences [47], changes in proportions of pre-frail and frail patients [53], and proportions of patients who changed frailty status [54]. To illustrate, Imhof et al. (2012), a fair quality article conducted in Switzerland, examined CGA conducted by advanced practice nurses at the homes of older persons aged ≥80, and reported that the intervention group had a lower relative risk of falls and prevalence of adverse consequences of falls at nine months follow-up [47]. Mazya et al. (2018) was a good quality study in Sweden, which investigated CGA conducted by a nurse and social worker at patients’ home and a pharmacist via the phone, for patients aged ≥75 years with three more chronic conditions and three or more inpatient admissions the past 12 months [53]. At 24 months follow-up, the intervention group had a significantly lower proportion of frail patients and significantly higher proportion of pre-frail patients, compared to controls [53]. Monteserin et al. (2010) was a good quality study conducted in Spain with CGAs conducted by nurses at a primary care health centre. The intervention group had a significantly lower proportion of persons who went from not at risk for frailty to at risk for frailty, and had a significantly greater proportion who maintained their frailty status, compared to controls, at 18 months follow-up [54].

The pre-post 12-week study based in the US by Faul et al. (2009) reported a significantly improved physical home environment and reduced fall hazards [44].

#### Mental health outcomes

Six RCTs [6, 35, 38, 40, 46, 57] and two pre-post studies [32, 44] examined the impact of CGA on mental health outcomes. Three of the six RCTs assessed depressive symptoms using the Geriatric Depression Scale (GDS) [57], psychological well-being and distress using Dupuy’s General Well-being Schedule (GWBS) [6] and general mental health with the 5-item Rand-36 mental health subscale [46], and showed no differences in outcomes.

The three RCTs that showed improved mental health assessed depressive symptoms with GDS [35] and Centre for Epidemiologic Studies- Depression scale (CES-D) [38], and mood and behaviour symptoms [40]. Boult et al. (2001), a good quality study, examined CGA conducted by nurses and a geriatrician at an ambulatory clinic at a community hospital in the US on older persons aged ≥70 years at a high risk of poor functional ability and high utilisation of health services, and revealed that the intervention group had significantly lower mean GDS scores for depressive symptoms at 18 months follow-up, compared to controls [35]. Burns et al. (2000) was a fair quality paper that examined CGA conducted by a multidisciplinary team in a primary care clinic in the US on persons aged ≥65 years with ADL deficits, chronic conditions, acute care hospitalisations the past year, and on scheduled prescriptions. The article reported greater improvement in the Centre for Epidemiologic Studies- Depression scale (CES-D) scores for depressive symptoms at two years’ follow-up in the intervention group, compared to controls [38]. Chi et al. (2006), a paper categorised as poor quality, investigated CGA conducted by trained interviewers at an elderly health centre in Hong Kong on Chinese older adults aged ≥65 years [40]. The study revealed greater reduction in the mean score for mood and behaviour symptoms at 12 months follow-up in the intervention group, compared to controls [40]. The two pre-post studies reported a lower mean score on the Cornell scale and 30-item GDS at three months follow-up [32], and a lower mean scores on the 15-item GDS at 12 weeks follow-up [44].

#### Self-rated health

Six RCTs and one pre-post study examined self-rated health. Only one of the six RCTs reported improved health perception, which was a two-year study in the US on CGA conducted by a multidisciplinary team in a geriatric primary care setting [38]. The pre-post article found improved self-rated health status at 12 weeks follow-up [44].

#### Cognition

Two RCTs [38, 40] and one pre-post study examined cognitive function [32]. Only one of the two RCTs found improved cognition in the intervention group compared to controls, and reported significantly improved Mini-Mental State Examination (MMSE) scores [38]. The pre-post study reported significant improvement in behavioural status among those with cognitive dysfunction, but among all participants, there were no differences in MMSE, clock drawing test and clinical dementia rating scale scores [32].

#### Chronic condition outcomes

Four RCTs and two pre-post studies investigated chronic condition outcomes. One of the four RCTs reported worse chronic condition outcomes, whereby the intervention group had poorer bowel incontinence compared to controls at three months follow-up, but there were no differences between groups for pain symptoms, urinary incontinence, and pressure ulcer [40]. Both pre-post studies found no difference in cumulative illness rating scale and cumulative illness rating scale comorbidity at three months follow-up [32], and no difference in self-efficacy for chronic disease management at 12 weeks follow-up [44].

#### Medication-related outcomes

One RCT [56], one case-control [45], and two pre-post studies [48, 52] examined medication-related outcomes. The RCT reported improved medication appropriateness assessed by the Medication Appropriateness Index and Assessment of Underutilisation in the intervention group, compared to controls, at 24 weeks follow-up [56]. The two pre-post studies revealed significant pre-post reduction in the proportion of patients with polypharmacy at 5.1 months follow-up, and significant pre-post reduction in the number of medications taken at 12 months follow-up [48, 52]. The case-control study found no significant difference in the rate of high-risk prescriptions at 20 months [45].

#### Nutritional status

Two pre-post studies examined nutritional status, and both found CGA to be significantly associated with a lower risk of malnutrition or being malnourished, measured using the Mini-Nutritional Assessment (MNA) scale [32, 48].

#### Quality of life outcomes

Seventeen RCTs [13, 19, 20, 34, 36, 37, 39–42, 46, 47, 51, 55, 56, 59, 61] and three pre-post studies [32, 48, 52] investigated the outcome on QoL. Twelve of the 17 RCTs found CGAs to have no significant impact on QoL [13, 20, 34, 36, 37, 42, 46, 47, 55, 56, 59, 61], while four RCTs reported improvements in QoL outcomes [19, 39, 41, 51]. Byles et al. (2004) revealed that both the SF-36 physical component and mental component summary scores improved in the intervention group compared to controls after three-years of follow-up [39]. The intervention involved home-based CGA by allied health professionals, verbal and written feedback, provision of printed health materials, and collaboration with general practitioners on follow-up matters [39]. In contrast, Cohen et al. (2002) reported that SF-36 scores for energy, general health, and mental health improved in the intervention group compared to controls after a year of follow-up [41]. The intervention involved CGA at an outpatient geriatric clinic by an geriatrician, nurse and social worker, followed by the coordination of preventive and management services based on individualised care needs [41]. Only Liimata et al. (2019) continued to assess QoL via the 15-dimensional assessment scale after the intervention, and found that even though the intervention group had significantly less decline in QoL at one year follow-up, after the home visits stopped, there was no longer any difference between groups at the end of the second year [51]. All three pre-post studies reported improved QoL using the EuroQoL Visual Analog Scale [32] EQ-5D [48] and a combination of quality-adjusted life years (QALY) and EQ-5D [52].

#### Mortality outcomes

Fourteen RCTs [19, 20, 35, 36, 38, 39, 41–43, 51, 53, 54, 56, 60], one controlled pre-post [49], and one case-control study [45] examined mortality. Only one of the 14 RCTs reported a significant reduction in risk of mortality at 36 months follow-up [43].

### Qualitative implementation barriers and facilitators

#### CGA implementation barriers and facilitators

Three qualitative studies [62–64] and four mixed-methods studies reported on the implementation barriers and benefits of CGA [65–68]. Five of these seven studies examined the implementation experience of healthcare professionals [62, 63, 65, 66, 68]. A different combination of five of the seven studies reported on the perspectives of patients [63–67].

#### Barriers

Barriers to implementation of CGAs can be categorised according to three key themes: lack of partnership alignment and feedback, poor acceptance of preventive work, and challenges in operationalising and optimising CGAs.

#### Lack of partnership alignment and feedback

When multi-agency teams were involved in the geriatric assessment and the delivery of care, differences in organisational culture, mental models of the service, varying expectations of job responsibilities [66, 68] and the risk of duplication of work, affected the formation and sustainability of partnerships [63, 65, 66]. A lack of direct communication between staff carrying out the geriatric assessment and general practitioners created cynicism about the relevance of the assessment efforts to care [62].

#### Poor acceptance of preventive work

Healthcare professionals faced difficulties in persuading patients of the preventive benefits of the service [62], and in creating trust and willingness to engage with the new service [65, 66]. Patients often did not hold the same definition of health problems, and may not perceive preventive services to be of value to them, especially for individuals who only required informational support on local services [64, 66].

#### Challenges in operationalising and optimising CGA

The planning and conduct of the geriatric assessment, and the process of following through on the findings [63, 68] were hampered by several factors. Variability in the duration of home visits and the lack of full reimbursement by payers affected the planning and initiation of home-based geriatric assessments [68]. As the domains of the CGA were predefined, patients faced difficulties in raising and discussing problems that fell outside its scope [64]. Trying to correctly time the CGA to meet the patients’ needs can be a challenge as the health status of older persons could change rapidly and unexpectedly [64].

To support the assessment of patients with multiple medical problems, the lack of local geriatrician support was a problem [65]. Studies also reported issues related to the reliability, format and ease of use of the CGA tool itself [62, 68], and the accuracy of the assessed domains [65]. For a more efficient coordination of and access to services, a comprehensive overview of local health and well-being facilities [65], and better social system support were required [68].

#### Facilitators

Perceived benefits of CGA that served to facilitate its implementation can be categorised into three themes: holistic assessment and education, highly skilled staff, and improvements in care coordination and convenience.

#### Holistic assessment and education

The CGA is perceived to facilitate an accurate assessment and discussion of a patient’s needs, including previously undetected and unreported health issues [62, 68]. Home-based CGA in particular, was reported to allow for direct observation and provided important information on an older person’s living environment and daily functioning, which in turn supported more detailed assessments [62, 63, 65, 68]. With additional manpower resources dedicated to the conduct of geriatric health assessments, healthcare professionals were able to educate at-risk patients to take responsibility of their health and provide practical advice on self-management [63].

#### Highly skilled staff

Skilful staff facilitated the implementation of geriatric health assessments [63, 64, 67]. Personal attributes such as being attentive and reassuring to the older person were part of this theme [64, 67]. Thorough explanation by the staff regarding the patient’s medical, psychosocial, and functional condition helped to improve their health literacy level [63], and the likelihood that the older adults would adopt the recommended services [63]. For complex cases, the staff’s ability to anticipate the patient’s needs and to coordinate care effectively across providers were greatly valued by the patients [63].

#### Improvements in care coordination and convenience

There was general agreement across the studies that the CGA gave rise to timely recommendations to services that helped to bridge previously unaddressed needs [62, 65, 66]. One study reported improved coordination of care, and reduced care fragmentation [63]. From the patient’s viewpoint, home-based assessment and home visits by the same care team provided convenience and continuity in care as it reduced the need to prepare or arrange for a clinic visit [67].

## Discussion

We conducted an integrative review to investigate the quantitative health outcomes and qualitative implementation barriers and facilitators of conducting CGA for older persons in the community setting. There were mixed findings for health outcomes. CGA in the community is a complex intervention, whereby the contexts, patients and the healthcare team interact to result in the mixed findings seen in this systematic review. The mixed evidence on health outcomes may reflect the variations in the implementation of CGA and target populations. For instance, selected studies had a range of settings where CGA was conducted, including the home, primary care, home and primary care, secondary or tertiary care setting, and geriatric clinic. CGA was conducted by different combinations of personnel, such as nurse only, geriatrician only, nurses and geriatricians, nurse and other allied health staff, and a multidisciplinary team. Additionally, selected articles had differences in study designs, length of follow up, methods of data collection and target population. Based on our qualitative findings, the implementation of the CGA required partnerships to be formed between different healthcare providers. This is challenging to sustain within fragmented health and social care systems where there is little acceptance of preventive care by the older patient population. Nonetheless, skilled staff can bring value through accurate assessment, empathetic communication, and coordination of services for community-based CGAs. The realist review on CGA in care homes by Chadborn et al. (2019) also asserted that successful CGA implementation required three main components, including structured comprehensive assessment, developing a care plan and working towards patient-centred goals [69]. This realist review and our qualitative findings in this study concur that CGA interventions focused only on assessment may not be effective in the long term [69]. Attention must be given to factors beyond care planning and assessment of needs.

As this is the first review in the literature on CGA in a community setting [3, 7–10], comparison of findings with existing reviews is limited. While we reported mixed findings on functional status, systematic reviews in inpatient settings have found improved functional status. A meta-analysis of RCTs on CGA for older adults admitted to hospitals reported significantly reduced deterioration in functional ability based on pooled estimates [8]. Another systematic review by Graf et al. (2011) concluded that CGA in the emergency department was efficient for decreasing functional decline [9]. A systematic review on CGA in primary care by Garrard et al. (2020) reported no improvements in functional outcomes based on one selected article [4]. In contrast to the mixed effects of CGAs conducted in community-based settings, we posit that CGA in inpatient settings are associated with improved functional status as patients admitted to the hospital or the emergency department likely comprised a subgroup of older adults who had multiple and more severe comorbid conditions and worse frailty status [70, 71]. These patients may have a higher potential for achieving significantly improved physical health outcomes after careful assessments with a clear plan for meeting their unmet needs. On the other hand, community-dwelling older adults are likely healthier with fewer unmet health and social needs, which may have limited the short-term impact of a CGA. We speculate that a longer follow-up time is needed to improve functional ability vis-a-vis a comparator group.

While we found some evidence suggesting that CGAs are associated with improved cognition, results on cognition reported by systematic reviews on CGA in inpatient settings are mixed. For instance, there was no change in cognition from CGA on older persons admitted to a hospital based on five RCTs categorised as low quality [7], and frail patients discharged from acute hospitals [3]. However, a meta-analysis of RCTs by Ellis et al. (2011) of CGA for older adults admitted to hospitals revealed that patients in the CGA intervention were significantly more likely to experience improved cognition [8]. Our findings on the lack of evidence that CGA reduced mortality is consistent with systematic reviews on CGA on patients admitted to a hospital [7], discharged from acute hospitals [3], and in primary care [4]. In contrast, Ellis et al. (2011) reported that CGA conducted on patients who were admitted to hospitals had higher odds of being alive compared to those who had general medical care, based on the synthesis of 11 trials [8].

Similar to our findings, other systematic reviews have also found inconclusive evidence on frailty and fall-related outcomes, and QoL outcomes. For instance, Conroy et al. (2011) reported inconclusive results on the benefits of CGA for frail older persons who were discharged from acute hospitals, and frailty and fall outcomes were not specifically studied [3]. A systematic review on CGA for patients admitted to hospitals found a small but significant increase in QALY based on three trials categorised as low quality [7]. Another systematic review on CGA for frail patients discharged from acute hospitals also revealed no change in QoL [3]. This review revealed that CGA in the community was associated with improved medication-related outcomes, which is consistent with the systematic review on CGA in primary care by Garrard et al. (2020) that reported evidence of better adherence to medication modifications [4].

Comparison of our qualitative findings with existing literature is also limited due to this being the first systematic review on CGA for community-dwelling older adults. Our qualitative results share some similarities with findings on CGA in acute settings. Westgård et al. (2019) conducted qualitative analyses on the experiences of frail older persons with CGA in an acute geriatric ward [22]. The authors revealed that older adults in a CGA-practising acute geriatric ward experienced receiving attention from medical staff which made them feel calm and safe, similar to our findings on how attentiveness of and reassurance from medical staff facilitated the implementation of CGA in the community [22]. Darby et al. (2017) examined the perspectives of patients who received CGA in an acute medical unit [21]. The article reported that older adults receiving CGA in acute settings also felt that they were merely being monitored and that CGA did not constitute active treatment [21], which is consistent with our findings on the lack of acceptance of CGA among community-dwelling older adults. The authors also found that older adults felt that CGA in acute settings did not support their on-going health and ADL needs post-discharge in time [21], which may be similar to one of the barriers found in the review on the difficulty of timing CGA in the community to meet patients’ needs due to the nature of older adults’ changing health status. Nonetheless, CGA in the community was generally perceived to facilitate timely recommendations to services that helped to bridge previously unaddressed needs.

Our review allows some discussion on the integration of quantitative and qualitative findings. For instance, the challenge of effective collaboration and operationalising CGA among healthcare providers, coupled with the lack of acceptance by patients, may have contributed to the underutilisation of certain health services and lack of improvement in health outcomes. This is consistent with studies that reported inadequate use of specific services such as mental health services [72]. Our qualitative findings on patients not having the same perceptions of health issues and facing difficulties discussing problems that fall outside the scope of pre-defined CGA domains, may have limited the positive effects on self-perceived outcomes like self-reported health and QoL. Additionally, patients may have unmet health needs (e.g., functional decline, frailty and falls, poor nutritional status) that stem from their perception that CGA has no preventive value, and that it is difficult for providers to time the CGA in line with patients’ changing needs. Studies have also shown that practice-based interventions for community-dwelling older persons had suboptimal implementation or processes. For instance, only a minority of patients with fear of falling had gait or balance evaluated, and a larger proportion of patients were examined for falls more intensively only after fall events [73]. Lastly, long-term commitment towards the implementation of recommendations is required for most health outcomes to achieve positive effects. The lack of communication between professionals conducting the CGA with general practitioners and geriatricians may impact efficient long-term follow-up with patients, and the lack of trust and willingness of patients to engage with providers may reduce adherence to care plans.

A strength of this study was the application of PROSPERO guidelines and the PRISMA flowchart to conduct a rigorous review. Quality assessments were conducted for specific study designs. We also applied an extensive search using medical and health sciences electronic databases, and precise search terms and inclusion criteria. However, this study has some limitations. First, we could not obtain full texts of three articles despite attempts to contact the authors and two papers were excluded as they were not in English, and these papers may have added further insights. Second, there were limitations in comparability between studies and drawing definitive conclusions due to the heterogeneity of interventions settings across studies, including age and selection of sample populations, where CGA was conducted, who conducted the CGA, follow-up duration, and tools used to measure outcomes. CGA in the community requires complex interventions in health and social care delivery, and heterogeneity of the literature would likely limit the reproduction of implementation strategies. Third, selected articles did not report on all the health outcomes, which limited the evidence base for each health outcome measure. However, there were still relatively high numbers of articles for outcomes on functional status, frailty and falls, quality of life, and mortality. While both physical and psychosocial health were commonly evaluated as part of the CGA which is intended to be a multidomain assessment of bio-psycho-social needs, most selected articles had their overall objective of the intervention to be on improving physical health like functional ability, frailty status, and mortality rates. Additionally, components such as financial health status were not evaluated, which may limit further insights on the impact on health outcomes. Fourth, we have synthesised findings on barriers and facilitators of CGA across studies situated within different policy, organisational and operational contexts. Despite this limitation, we believe there is still merit in summarising a set of commonly occurring themes that have influenced the implementation of CGAs to inform future efforts. Lastly, our search strategy did not include broader terms, such as “integrated care”. We believe that including a broad term like “integrated care” will substantially increase the number of citations for screening and resources needed, without adding to the number of relevant articles that meet the inclusion criteria. The selected articles had to meet precise criteria on the CGA intervention, and specifically evaluate its impact on health outcomes and/or report barriers and facilitators. Articles on integrated care that fulfilled these criteria will likely include the keywords and phrases in our search strategy in their titles and abstracts. We balanced our ability to maximise obtaining relevant articles with the need to not overuse our limited time and resources.

We have seven recommendations on implementing CGA in the community. First, we recommend having other interventions in conjunction to CGA. For instance, other modalities like physical activity interventions may augment the effects of CGA for frailty progression, especially in pre-frail and frail populations [74, 75]. Second, the mixed evidence in this review makes it challenging to propose practice recommendations. There is currently a lack of clarity in the literature on subgroup level differences that impact CGA effectiveness. This includes target populations, settings (home, primary care) and personnel conducing CGA. We recommend practitioners to start new CGA programs in the community in a conservative manner. For these new community CGA programs, we suggest implementing CGA for subgroups that are more likely to benefit, instead of carrying out broad-based CGA programs. Expansion of CGA in the community could be done conservatively. Further research and evidence are needed to better understand the mechanism of change prior to expanding community CGA to wider groups of older adults.

Third, our qualitative findings suggest that patients do not value preventative services. Hence healthcare staff need to be intentional in informing patients of the preventive benefits of CGA. Fourth, as CGA may be a new concept to older adults, there can be health programs to increase the awareness of CGA. To increase the uptake of CGA by patients, CGA could be more accessible by improving administrative processes and financial support. Fifth, another qualitative finding is on the challenges with operationalising CGA across agencies. We recommend multi-agency teams to establish clearer operating procedures to facilitate better partnerships, and minimise misunderstandings of job expectations and duplication of work. Parties involved should intentionally develop of a system that engages multi-agency staff, facilitates operation under different occupational goals and cultures, and develop shared practice-oriented outcomes. Next, our qualitative findings highlight that the patients do not perceive CGA to be useful when the only information they receive is on follow-up services needed. Older adults usually require subsequent follow up by multiple stakeholders to meet their needs that were identified from CGA. There could be an operating protocol to differentiate older adults who only need to be directed to personnel who can provide information on services, from those who require further assessment and treatment. Lastly, a recommendation is for healthcare staff to be more open to discussing patients’ problems that fall outside the scope of the CGA domains. This is to address the problem of pre-defined CGA domains that discouraged patients from discussing other issues.

We also propose some recommendations for future research. First, a knowledge gap in the current literature is the lack of studies conducted outside the US and Europe. Future research is needed in other parts of the world. We hypothesise that the effects of CGA in the community in the US and Europe and other parts of the world will have differences, due to different healthcare systems, health policies and infrastructures. Second, we need to better examine whether community-based CGA could be beneficial for certain subgroups, such as those with frailty, of highly advanced age, functional impairments, or transitioning between care settings. Third, to shift care away from inpatient settings and expand preventive care in the community, more research is needed on the implementation of CGA in primary care and comparing its effects with other community settings. Fourth, as a minority of articles had CGA conducted by geriatricians, future research could examine whether having a geriatrician within the healthcare team that conducts the CGA has differential effects on health outcomes. Fifth, drawing from both qualitative and quantitative findings, the question of whether poor implementation led to the lack of positive effects or if CGA was ineffective is unanswered. Hence, further mixed methods studies could tease out the mechanisms of change. Lastly, this review did not reveal notable differences in CGA conducted in rural versus urban areas. However, future research could examine the implications of implementing CGA in rural areas, where there are challenges in health system performance and access to healthcare resources [76, 77].

## Conclusion

There is mixed evidence on the quantitative health outcomes of CGA on community-dwelling older adults. There is a need to better understand whether the current heterogeneity in effects and lack of positive findings persist across all groups of patients or if certain subgroups could still benefit from community-based CGA. Based on our qualitative results, we found that even though there is value from CGA when carried out by highly skilled staff, barriers such as bringing providers into a partnership, greater acceptance of preventive care, and operational issues could impede its implementation.

## Supplementary Information

**Additional file 1.**

**Additional file 2.**

**Additional file 3.**

## Data Availability

The datasets used and/or analysed during the current study are available from the corresponding author on reasonable request.
