# Supplementary material for: Health outcomes and implementation barriers and facilitators of comprehensive geriatric assessment in community settings: a systematic integrative review [PROSPERO registration no.: CRD42021229953]
Source: BMC Geriatr. 2022 Apr 29;22:379. doi: 10.1186/s12877-022-03024-4 (PMC9052611; doi:10.1186/s12877-022-03024-4)
Supplement: Supplementary file 1 — Additional file 1. [file 12877_2022_3024_MOESM1_ESM.docx]

**Appendix A.** Search strategy for each electronic database

The date limit was added after downloading the citations.

Database: Ovid Medline

| **Step** | **Search input** |
| --- | --- |
| 1 | Geriatric Assessment/ or Needs Assessment/ |
| 2 | ("geriatric evaluation?" or "geriatric assessment?" or (comprehensive adj3 assessment) or "geriatric consultation").ab,ti. |
| 3 | 1 or 2 |
| 4 | exp Aged/ |
| 5 | (aged or senior? or "older adult?" or elderly or elder?).ab,ti. |
| 6 | 4 or 5 |
| 7 | exp General Practice/ or physicians, family/ or physicians, primary care/ or Primary Health Care/ or Community Health Centers/ or Independent Living/ or Ambulatory Care/ |
| 8 | ("general practice" or "family practice" or "family physician?" or "primary care physician?" or "primary care" or "primary health care" or "primary healthcare" or "community health centre?" or "community health center?" or "independent living" or "aging in place" or community or "ambulatory care" or "outpatient clinic?" or "outpatient health service?" or "outpatient care" or "outpatient service?" or "day care" or "day rehabilitation").ab,ti. |
| 9 | 7 or 8 |
| 10 | 3 and 6 and 9 |
| 11 | limit 10 to English language |

Database: EMBASE

| **Step** | **Search input** |
| --- | --- |
| 1 | geriatric assessment/ or needs assessment/ |
| 2 | ("geriatric evaluation?" or "geriatric assessment?" or (comprehensive adj3 assessment) or "geriatric consultation").ab,ti. |
| 3 | 1 or 2 |
| 4 | exp aged/ |
| 5 | (aged or senior? or "older adult?" or elderly or elder?).ab,ti. |
| 6 | 4 or 5 |
| 7 | general practice/ or general practitioner/ or exp primary health care/ or health center/ or independent living/ or ambulatory care/ |
| 8 | ("general practice" or "family practice" or "family physician?" or "primary care physician?" or "primary care" or "primary health care" or "primary healthcare" or "community health centre?" or "community health center?" or "independent living" or "aging in place" or community or "ambulatory care" or "outpatient clinic?" or "outpatient health service?" or "outpatient care" or "outpatient service?" or "day care" or "day rehabilitation").ab,ti. |
| 9 | 7 or 8 |
| 10 | 3 and 6 and 9 |
| 11 | limit 10 to English language |

Database: Cumulative Index of Nursing and Allied Health Literature (CINAHL)

| **Step** | **Search input** |
| --- | --- |
| 1 | (MH "Needs Assessment") OR (MH "Geriatric Assessment+") |
| 2 | TI ("geriatric evaluation#" or "geriatric assessment#" or (comprehensive N3  assessment) or "geriatric consultation" ) OR AB ("geriatric evaluation#" or  "geriatric assessment#" or (comprehensive N3 assessment) or "geriatric  consultation") |
| 3 | S1 OR S2 |
| 4 | (MH "Aged") |
| 5 | TI (aged or senior# or "older adult#" or elderly or elder# ) OR AB ( aged  or senior# or "older adult#" or elderly or elder#) |
| 6 | S4 OR S5 |
| 7 | (MH "Family Practice") OR (MH "Physicians, Family") OR (MH "Primary Health Care") OR (MH "Community Health Centers") OR (MH "Community Living")  OR (MH "Ambulatory Care") |
| 8 | TI ("general practice" or "family practice" or "family physician#" or  "primary care physician#" or "primary care" or "primary health care" or "primary  healthcare" or "community health centre#" or "community health center#" or  "independent living" or "aging in place" or community or "ambulatory care" or  "outpatient clinic#" or "outpatient health service#" or "outpatient  care" or "outpatient service#" or "day care" or "day rehabilitation") OR  AB ("general practice" or "family practice" or "family physician#" or  "primary care physician#" or "primary care" or "primary health care" or "primary  healthcare" or "community health centre#" or "community health center#" or  "independent living" or "aging in place" or community or "ambulatory care" or  "outpatient clinic#" or "outpatient health service#" or "outpatient care" or "outpatient service#" or "day care" or "day rehabilitation") |
| 9 | S7 OR S8 |
| 10 | S3 AND S6 AND S9 |
| 11 | S3 AND S6 AND S9 |
| 12 | S3 AND S6 AND S9 |

Database: PsycINFO

| **Step** | **Search input** |
| --- | --- |
| 1 | (DE "Geriatric Assessment") OR (DE "Needs Assessment") |
| 2 | TI ("geriatric evaluation#" or "geriatric assessment#" or (comprehensive N3  assessment) or "geriatric consultation" ) OR AB ("geriatric evaluation#" or  "geriatric assessment#" or (comprehensive N3 assessment) or "geriatric  consultation") |
| 3 | S1 OR S2 |
| 4 | TI (aged or senior# or "older adult#" or elderly or elder#) OR AB (aged  or senior# or "older adult#" or elderly or elder#) |
| 5 | (((DE "Family Physicians") OR (DE "Primary Health Care")) OR (DE "Self-Care  Skills")) OR (DE "Outpatient Treatment") |
| 6 | TI ("general practice" or "family practice" or "family physician#" or  "primary care physician# "or "primary care" or "primary health care" or "primary healthcare" or "community health centre#" or "community health center#" or "independent living" or "aging in place" or community or "ambulatory care" or  "outpatient clinic#" or "outpatient health service#" or "outpatient care" or "outpatient service#" or "day care" or "day rehabilitation") OR AB ( "general practice" or "family practice" or "family physician#" or "primary care physician#"  or "primary care" or "primary health care" or "primary healthcare" or  "community health centre#" or "community health center#" or "independent living" or "aging in place" or community or "ambulatory care" or "outpatient clinic#" or "outpatient health service#" or "outpatient care" or "outpatient  service#" or "day care" or "day rehabilitation") |
| 7 | S5 OR S6 |
| 8 | S3 AND S4 AND S7 |
| 9 | S3 AND S4 AND S7 Narrow by Language: - English |
