# Supplementary material for: Health outcomes and implementation barriers and facilitators of comprehensive geriatric assessment in community settings: a systematic integrative review [PROSPERO registration no.: CRD42021229953]
Source: BMC Geriatr. 2022 Apr 29;22:379. doi: 10.1186/s12877-022-03024-4 (PMC9052611; doi:10.1186/s12877-022-03024-4)
Supplement: Supplementary file 2 — Additional file 2. [file 12877_2022_3024_MOESM2_ESM.docx]

**Appendix B.** List of quality evaluation questions for each type of study design

**Controlled intervention studies**

**Options:** Yes, No, Cannot be determined, Not reported, Not applicable

Question 1. Was the study described as randomized, a randomized trial, a randomized clinical trial, or an RCT?

Question 2. Was the method of randomization adequate (i.e., use of randomly generated assignment)?

Question 3. Was the treatment allocation concealed (so that assignments could not be predicted)?

Question 4. Were study participants and providers blinded to treatment group assignment?

Question 5. Were the people assessing the outcomes blinded to the participants' group assignments?

Question 6. Were the groups similar at baseline on important characteristics that could affect outcomes (e.g., demographics, risk factors, co-morbid conditions)?

Question 7. Was the overall drop-out rate from the study at endpoint 20% or lower of the number allocated to treatment?

Question 8. Was the differential drop-out rate (between treatment groups) at endpoint 15 percentage points or lower?

Question 9. Was there high adherence to the intervention protocols for each treatment group?

Question 10. Were other interventions avoided or similar in the groups (e.g., similar background treatments)?

Question 11. Were outcomes assessed using valid and reliable measures, implemented consistently across all study participants?

Question 12. Did the authors report that the sample size was sufficiently large to be able to detect a

difference in the main outcome between groups with at least 80% power?

Question 13. Were outcomes reported or subgroups analyzed prespecified (i.e., identified before analyses were conducted)?

Question 14. Were all randomized participants analyzed in the group to which they were originally assigned, i.e., did they use an intention-to-treat analysis?

**Pre-post without controls**

**Options:** Yes, No, Cannot be determined, Not reported, Not applicable

Question 1. Was the study question or objective clearly stated?

Question 2. Were eligibility/selection criteria for the study population prespecified and clearly described?

Question 3. Were the participants in the study representative of those who would be eligible for the test/service/intervention in the general or clinical population of interest?

Question 4. Were all eligible participants that met the prespecified entry criteria enrolled?

Question 5. Was the sample size sufficiently large to provide confidence in the findings?

Question 6. Was the test/service/intervention clearly described and delivered consistently across the study population?

Question 7. Were the outcome measures prespecified, clearly defined, valid, reliable, and assessed consistently across all study participants?

Question 8. Were the people assessing the outcomes blinded to the participants' exposures/interventions?

Question 9a. Was the loss to follow-up after baseline 20% or less?

Question 9b. Were those lost to follow-up accounted for in the analysis?

Question 10a. Did the statistical methods examine changes in outcome measures from before to after the intervention?

Question 10b. Were statistical tests done that provided p values for the pre-to-post changes?

Question 11. Were outcome measures of interest taken multiple times before the intervention and multiple times after the intervention (i.e., did they use an interrupted time-series design)?

Question 12. If the intervention was conducted at a group level (e.g., a whole hospital, a community, etc.) did the statistical analysis take into account the use of individual-level data to determine effects at the group level?

**Case controls**

**Options:** Yes, No, Cannot be determined, Not reported, Not applicable

Question 1. Was the research question or objective in this paper clearly stated and appropriate?

Question 2. Was the study population clearly specified and defined?

Question 3. Did the authors include a sample size justification?

Question 4. Were controls selected or recruited from the same or similar population that

gave rise to the cases (including the same timeframe)?

Question 5. Were the definitions, inclusion and exclusion criteria, algorithms or processes used to identify or select cases and controls valid, reliable, and implemented consistently across all study participants?

Question 6. Were the cases clearly defined and differentiated from controls?

Question 7. If less than 100 percent of eligible cases and/or controls were selected for the study, were the cases and/or controls randomly selected from those eligible?

Question 8. Was there use of concurrent controls?

Question 9. Were the investigators able to confirm that the exposure/risk occurred prior to the development of the condition or event that defined a participant as a case?

Question 10. Were the measures of exposure/risk clearly defined, valid, reliable, and implemented consistently (including the same time period) across all study participants?

Question 11. Were the assessors of exposure/risk blinded to the case or control status of participants?

Question 12a. Were key potential confounding variables measured and adjusted statistically in the analyses?

Question 12b. If matching was used, did the investigators account for matching during study analysis?

**Qualitative**

**Options:** Yes, No, Can’t tell

Question 1. Was there a clear statement of the aims of the research?

Question 2. Is a qualitative methodology appropriate?

Question 3. Was the research design appropriate to address the aims of the research?

Question 4. Was the recruitment strategy appropriate to the aims of the research?

Question 5. Was the data collected in a way that addressed the research issue?

Question 6. Has the relationship between researcher and participants been adequately considered?

Question 7. Have ethical issues been taken into consideration?

Question 8. Was the data analysis sufficiently rigorous?

Question 9. Is there a clear statement of findings?

Question 10. How valuable is the research?

**Mixed methods**

**Options:** Yes, No, Can’t tell

Question 1. Is there an adequate rationale for using mixed methods design to address the research question?

Question 2. Are the different components of the study effectively integrated to answer the research question?

Question 3. Are the outputs of the integration of qualitative and quantitative components adequately interpreted?

Question 4. Are divergences and inconsistencies between quantitative and qualitative results adequately addressed?

Question 5. Do the different components of the study adhere to the quality criteria of each tradition of the methods involved?

**Appendix C.** Quality evaluation scores for each type of study design

**Summary of quality evaluation results**

| **Quality**  **N (%)** | **Study design** | | | | | |
| --- | --- | --- | --- | --- | --- | --- |
|  | Controlled intervention studies  (n=31) | Pre-post without controls  (n=4) | Case-control studies  (n=1) | Qualitative studies  (n=3) | Mixed methods studies  (n=4) | Total  (n=43) |
| Good | 19 (61.3) | 0 | 1 (100%) | 2 (66.7%) | 1 (25.0%) | 23 (53.5%) |
| Fair | 9 (29.0) | 4 (100%) | 0 | 1 (33.3%) | 2 (50.0%) | 16 (37.2%) |
| Poor | 3 (9.7) | 0 | 0 | 0 | 1 (25.0%) | 4 (9.3%) |

**Controlled intervention studies**

| **S/N** | **Reference** | **Q1** | **Q2** | **Q3** | **Q4** | **Q5** | **Q6** | **Q7** | **Q8** | **Q9** | **Q10** | **Q11** | **Q12** | **Q13** | **Q14** | **Total Score** | **Quality category** |
| --- | --- | --- | --- | --- | --- | --- | --- | --- | --- | --- | --- | --- | --- | --- | --- | --- | --- |
| 1 | Avlund et al 2002 | Y | Y | Y | N | Y | NR | NR | NR | NR | NR | Y | N | NR | Y | 6 | Poor |
| 2 | Bleihenberg et al 2017 | Y | Y | Y | N | N | Y | Y | Y | NR | N | Y | N | Y | Y | 9 | Fair |
| 3 | Blom et al 2016 | Y | Y | Y | N | Y | Y | Y | Y | NR | NR | Y | Y | Y | Y | 11 | Good |
| 4 | Boult et al 2001 | Y | CD | Y | N | Y | Y | Y | Y | NR | NR | Y | N | Y | Y | 9 | Fair |
| 5 | Boult et al 2013 | Y | Y | Y | N | Y | N | N | Y | NR | NR | Y | Y | Y | Y | 10 | Good |
| 6 | Bouman et al 2008 | Y | Y | Y | N | Y | Y | Y | Y | N | Y | Y | Y | NR | Y | 11 | Good |
| 7 | Burns et al 2000 | Y | NR | N | N | N | Y | Y | Y | NR | Y | Y | NR | Y | Y | 8 | Fair |
| 8 | Byles et al 2004 | Y | Y | Y | N |  | Y | N | Y | NR | Y | Y | Y | NR | Y | 10 | Good |
| 9 | Chi et al 2006 | Y | CD | NR | NR | NR | CD | N | Y | NR | NR | Y | CD | Y | Y | 5 | Poor |
| 10 | Cohen et al 2002 | Y | Y | Y | N | Y | Y | Y | Y | NR | Y | Y | Y | NR | N | 10 | Good |
| 11 | Eckerblad et al 2016 | Y | Y | Y | N | NR | Y | Y | Y | NR | Y | Y | N | NR | Y | 9 | Fair |
| 12 | Ekdahl et al 2015 | Y | Y | Y | N | Y | Y | Y | Y | NR | Y | Y | Y | Y | Y | 12 | Good |
| 13 | Ekdahl et al 2016 | Y | Y | Y | N | Y | Y | Y | Y | Y | Y | Y | Y | N | Y | 12 | Good |
| 14 | Fletcher et al 2004 | Y | Y | Y | N | Y | Y | N | Y | NR | Y | Y | Y | Y | Y | 11 | Good |
| 15 | Godwin et al 2016 | Y | NR | NR | N | N | Y | N | Y | NR | Y | Y | N | NR | Y | 6 | Poor |
| 16 | Hebert et al 2001 | Y | Y | Y | N | NR | Y | Y | Y | NR | No | Yes | Yes | Yes | Yes | 10 | Good |
| 17 | Hoogendijk 2016 | Y | Y | Y | N | NR | N | Y | Y | Y | Y | Y | Y | Y | Y | 11 | Good |
| 18 | Imhof et al 2012 | Y | Y | Y | N | N | Y | Y | Y | NR | Y | Y | N | NR | Y | 9 | Fair |
| 19 | King et al 2018 | N | NA | N | Y | N | Y | Y | Y | NR | Y | Y | CD | Y | Y | 7 | Fair |
| 20 | Li et al 2010 | Y | Y | Y | N |  | Y | Y | Y | N | Y | Y | N | N | N | 9 | Fair |
| 21 | Liimatta et al 2019 | Y | Y | NR | NR | NR | Y | N | Y | NR | NR | Y | Y | Y | Y | 8 | Fair |
| 22 | Mazya et al 2019 | Y | Y | Y | N | Y | Y | N | Y | NR | Y | Y | Y | N | Y | 10 | Good |
| 23 | Monteserin et al 2010 | Y | Y | Y | N | Y | Y | Y | Y | NR | Y | Y | Y | CD | Y | 11 | Good |
| 24 | Ploeg et al 2010 | Y | Y | Y | N | Y | Y | Y | Y | NR | NR | Y | Y | Y | Y | 10 | Good |
| 25 | Romskaug et al 2020 | Y | Y | Y | N | Y | Y | Y | Y | NR | Y | Y | N | Y | Y | 11 | Good |
| 26 | Rubenstein et al 2007 | Y | Y | Y | N | Y | Y | N | Y | N | Y | Y | Y | NR | Y | 10 | Good |
| 27 | Stuck et al 2000 | Y | Y | Y | N | Y | Y | Y | Y | NR | Y | Y | Y | Y | Y | 12 | Good |
| 28 | Suijker et al 2016 | Y | Y | Y | N | Y | Y | N | Y | N | Y | Y | Y | Y | Y | 11 | Good |
| 29 | Suijker et al 2017 | Y | Y | Y | N | Y | Y | N | Y | N | Y | Y | Y | Y | Y | 11 | Good |
| 30 | van Hout et al 2010 | Y | Y | Y | N | Y | Y | N | Y | NR | Y | Y | N | Y | Y | 10 | Good |
| 31 | van Leeuwen et al 2015 | Y | Y | Y | N | CD | N | N | Y | NR | Y | Y | Y | Y | Y | 9 | Fair |

Y= Yes; N= No; CD= Cannot determine; NR= Not reported; NA= Not applicable

**Pre-post without controls**

| **S/N** | **Reference** | **Q1** | **Q2** | **Q3** | **Q4** | **Q5** | **Q6** | **Q7** | **Q8** | **Q9a** | **Q9b** | **Q10a** | **Q10b** | **Q11** | **Q12** | **Total Score** | **Quality category** |
| --- | --- | --- | --- | --- | --- | --- | --- | --- | --- | --- | --- | --- | --- | --- | --- | --- | --- |
| 1 | Ballabio et al 2008 | Y | Y | Y | NA | N | Y | N | N | Y | NR | Y | Y | N | NR | 7 | Fair |
| 2 | Faul et al 2009 | Y | Y | Y | NA | N | Y | Y | N | Y | N | Y | Y | N | Y | 9 | Fair |
| 3 | Kang et al 2020 | Y | Y | Y | NA | NR | Y | Y | N | Y | N | Y | Y | N | Y | 9 | Fair |
| 4 | Lin et al 2012 | Y | Y | Y | NA | NR | Y | N | N | Y | NR | Y | Y | NR | NR | 7 | Fair |

Y= Yes; N= No; CD= Cannot determine; NR= Not reported; NA= Not applicable

**Case-control studies**

| **S/N** | **Reference** | **Q1** | **Q2** | **Q3** | **Q4** | **Q5** | **Q6** | **Q7** | **Q8** | **Q9** | **Q10** | **Q11** | **Q12a** | **Q12b** | **Total Score** | **Quality category** |
| --- | --- | --- | --- | --- | --- | --- | --- | --- | --- | --- | --- | --- | --- | --- | --- | --- |
| 1 | Fenton et al 2006 | Y | Y | Y | N | Y | Y | NA | N | Y | Y | N | Y | Y | 9 | Good |

Y= Yes; N= No; CD= Cannot determine; NR= Not reported; NA= Not applicable

**Qualitative studies**

| **Article no.** | **Reference** | **Q1** | **Q2** | **Q3** | **Q4** | **Q5** | **Q6** | **Q7** | **Q8** | **Q9** | **Q10** | **Total Score** | **Quality category** |
| --- | --- | --- | --- | --- | --- | --- | --- | --- | --- | --- | --- | --- | --- |
| 1 | Byles et al 2002 | Y | Y | Y | Y | Y | CT | CT | CT | Y | The research investigates healthcare professionals’ experience with home assessments for older adults and discussed findings in relation to existing literature and policy. | 6 | Fair |
| 2 | King et al 2018 | Y | Y | Y | Y | Y | CT | CT | Y | Y | The research is discussed in context, taking into account the benefits, drawbacks and the applicability of implementing the intervention in the current system, and highlights the importance of it for frail older adults. | 7 | Good |
| 3 | Rietkerk et al 2019 | Y | Y | Y | Y | Y | Y | Y | Y | Y | The research examines patients’ experiences with different components of a comprehensive geriatric assessment programme which provides insight into why certain programmes yield ambiguous results, and elucidates relevant areas of improvement for care for older adults. | 9 | Good |

Y= Yes; N= No; CT= Can’t tell

**Mixed Methods studies**

| **S/N** | **Reference** | **Q1** | **Q2** | **Q3** | **Q4** | **Q5** | **Total Score** | **Quality category** |
| --- | --- | --- | --- | --- | --- | --- | --- | --- |
| 1 | Cravens et al 2005 | Y | N | Y | N | CT | 2 | Poor |
| 2 | Drennan et al 2005 | Y | Y | Y | Y | Y | 5 | Good |
| 3 | Fristedt et al 2019 | Y | Y | Y | N | CT | 3 | Fair |
| 4 | Stijnen et al 2013 | Y | Y | Y | Y | N | 4 | Fair |

Y= Yes; N= No; CT= Can’t tell
