# Supplementary material for: Health outcomes and implementation barriers and facilitators of comprehensive geriatric assessment in community settings: a systematic integrative review [PROSPERO registration no.: CRD42021229953]
Source: BMC Geriatr. 2022 Apr 29;22:379. doi: 10.1186/s12877-022-03024-4 (PMC9052611; doi:10.1186/s12877-022-03024-4)
Supplement: Supplementary file 3 — Additional file 3. [file 12877_2022_3024_MOESM3_ESM.docx]

**Appendix D.** Table on summary of quantitative health outcomes (n= 36)

| **Article no.** | **Reference, Quality** | **Setting** | **Study design, follow up duration, population** | **Comprehensive geriatric assessment (CGA)** | **Objective of intervention** | **Usual care** | **Quantitative health outcomes** | **Other outcomes**  **(healthcare utilisation, polypharmacy, cost)** |
| --- | --- | --- | --- | --- | --- | --- | --- | --- |
| 1 | Avlund et al 2002 | Home visits after hospitalisation  Denmark | RCT  3 months  32 persons aged >70 years at the medical wards. | At home  Conducted by: General practitioner, home nurse, home helper, physiotherapist, and/or occupational therapist.  Components: Health and medical problems. | To reduce readmissions and improve functional ability for older adults after discharge from hospital. | Existing norms for discharge planning were applied to all control patients. | Functional status  -Significant improvement in mean Barthel Index. | Healthcare utilisation  - No difference in readmission rates. |
| 2 | Ballabio et al 2008 | Outpatient geriatric unit  Italy | Pre-post design  3 months  222 persons aged ≥75 years who were discharged from the emergency department. | Outpatient geriatric unit.  Conducted by: Geriatrician, nurse and social worker.  Components: Physical status, functional status, cognitive status, depression, cognitive dysfunction, caregiver stress, perceived QoL. | To reduce risk of adverse health outcomes after discharge from the emergency department, and to reduce readmissions to the emergency department. | Not applicable (no controls) | Functional status  - No significant difference in ADL and IADL scores.  Quality of life  - Significant improved QoL via EuroQoL analogic section.  Mental health  - Significant improvement in emotional status, via the Cornell scale and GDS.  Cognition  - Significant improvement in behavioural status in those with cognitive dysfunction.  - Among all subjects, no significant change in MMSE scores, clock drawing test, and clinical dementia rating scale.  Chronic condition  - No significant difference in cumulative illness rating scale severity and cumulative illness rating scale comorbidity.  Nutritional status  - Significant improvement in mini-nutritional assessment scale. | Healthcare utilisation  - Significant reduction in readmission to the emergency department and hospitalisations.  Caregiver outcomes  - Significant reduction in distress in caregivers of patients with cognitive dysfunction and behavioural disturbances.  - No significant change in caregiver burden inventory measure. |
| 3 | Bleijenberg et al 2017 | Nurse-Led Care Programs  Switzerland and Netherlands | Pooled analysis of 2 RCTs: Utrecht Proactive Frailty Intervention Trail (UPROFIT), the Netherlands)**;** in home health consultation program (HCP), Switzerland  12 months for 230 subjects, 9 months for 231 subjects.  461 persons aged ≥80 years, whereby 230 from UPROFIT had multimorbidity, polypharmacy and care gap in primary care of ≥3 years, and 231 from HCP were from healthcare organisations, local hospitals and social services. | At home and primary care  Conducted by: Advance practice registered nurses.  Components: Clinical assessment of health and family situation, symptoms of illness, frailty and falls, urinary incontinence, cognition, loneliness. | To improve functional status and understand the factors that influence better functional status from a CGA nurse-led intervention. | Received healthcare services as usual provided by community health nurses and physicians. | Functional status  - 65.7% of participants from the HCP Trial, and 50.9% of  participants from the UPROFIT Trial had a successful response, defined as an improvement or stability in daily functioning.  - Having fewer comorbidities and a better self-rated health had the strongest predictive value for benefiting from the intervention (OR = 0.83 [95% CI 0.66–1.03], and OR = 1.5 [95% CI 0.92–2.45]), respectively. |  |
| 4 | Blom et al 2016 | General practice  Netherlands | RCT  1 year  59 practices with 7278 participants aged ≥75 years | General practice  Conducted by: General practitioner or practice nurse.  Components: Functional, somatic (health and illness), mental, and social Each domain contained 4–9 questions. | To improve functioning and personal quality of life by integrating healthcare providers. | Control practices had usual care without delivery of proactive, goal-oriented, integrated care. | Functional status  - No significant changes in GARS total score, GARS subscale BADL, BARS subscale.  Quality of life  - No significant changes in Cantril’s Ladder.  Mental health  - No significant change in Geriatric depression scale-15 (GDS-15) and self-rated loneliness via the Loneliness Scale of De Jong Gierveld (DJG). | Cost  Costs were estimated at €236 per care plan.  This cost constituted only 1.3% of the total healthcare costs during the 1-year follow-up. No differences were found in the use of other types of health  care or in total healthcare costs.  Satisfaction and meeting care needs  - No. of people satisfied with the general practitioner increased in the intervention group and decreased in the control group.  - No difference between the two groups was  observed on confidence in the GP or on satisfaction with and confidence in the other care providers.  - GPs reported an improvement in the overview of care needs. |
| 5 | Boult et al 2001 | Outpatient geriatric evaluation and management  United States | RCT  18 months  568 persons aged ≥70 years at a high risk of poor functional ability and high use of health services. | At an outpatient geriatric evaluation and management clinic  (ambulatory clinic) in a community hospital  Conducted by: General nurse practitioner, geriatrician, and nurse.  Components: Medical conditions, psychosocial status, functional ability, cognitive status, nutritional risk, use of alcohol, social network,  gait and balance, environmental safety, medications, advance directives, hearing, vision. | To reduce risk of poor functional ability and high use of health services. | Healthcare that patients and their physicians deemed appropriate. | Functional status  - Intervention group has significantly lower likelihood for reduced functional ability.  - Intervention group has significantly lower likelihood to have increased health-related restrictions in their daily activities.  Mental health  - Intervention group had significantly lower GDS scores for depression.  Mortality rate  - No difference between groups in mortality rate. | Healthcare utilisation  - Intervention group had a significantly lower likelihood of using any home care, compared to the controls.  Cost  - No significant difference between groups for total 18-month Medicare expenditures. |
| 6 | Boult et al 2013 | Guided care for high-risk older adults  United States | RCT  32 months  904 persons aged ≥65 years t high risk of using health services heavily during the following year, as estimated by the claims based hierarchical condition category (HCC) predictive model. | At home  Conducted by: registered nurses with at least 3 years of clinical experience and who took a Guided Care preparatory course.  Components: Not specified, but comprehensive assessments were conducted with individualised action plans designed. | To produce better functional health and quality of care, while reducing the use of expensive health services. | Usual care was continued care from established primary care physicians. | Quality of life  - No difference between groups in physical and mental scores  Mortality  - No difference between groups in mortality rate.  Health status  - No difference between groups in self-rated health. | Healthcare utilisation  - Intervention group significantly used a lower rate of home health care.  - No difference between groups in number of hospital admissions, 30-day hospital re-admissions, skilled nursing facility days, primary care visits, specialist visits, and emergency department visits.  Quality of care  - Intervention group had significantly higher quality of care of chronic conditions from Guided Care.  - Guided Care recipients were  more likely to report “excellent or very good” access to telephone advice. |
| 7 | Bouman et al 2008 | Home visiting program  Netherlands | RCT  18 months  293 persons aged 70 to 84 years who lives at home, and has poor health status. | At home  Conducted by: Home nurses (auxiliary community nurses)  under the supervision of public health nurses (community nurses).  Components: Health problems and risks. | To improve self-rated health, functional status, quality of life, and changes in self-reported problems. | Usual care without home visits, telephone follow up, health assessments, advice, and referrals to professional and community services. | Functional status  - No difference in ADL scores.  Quality of life  - No difference in quality of life.  Health status  - No difference in self-rated health score and self-reported health problems. |  |
| 8 | Burns et al 2000 | Geriatric Primary Care  United States | RCT  2 years  128 persons aged ≥65 years with ADL deficits, chronic conditions, acute care hospitalisations in previous year, and on scheduled prescriptions. | Geriatric primary care  Conducted by: Interdisciplinary care team.  Components: Health status including mortality, global health perception, clinic visits and hospitalisations,  functional status, global social activity, quality of life, life satisfaction, and  cognition. | To provide long-term primary care management to geriatrics. | Received usual care through the Veterans Affairs Ambulatory Clinics. | Functional status  - Intervention group had significantly lower increases in IADL impairments.  - No difference between groups for improvement in ADLs scores.  Mortality rate  - No difference between groups in mortality rate.  Mental health  - Intervention group had greater improvement in Center for Epidemiologic Studies-Depression scores, well-being and life satisfaction.  Health status  - Intervention group significantly greater improvement in health perception.  Cognition  - Intervention group significantly greater improvement in Mini-Mental State Examination score. | Healthcare utilisation  - Intervention group had significantly smaller increases in number of clinic visits.  - No difference between groups for hospitalisations.  Social support  - Intervention group had significantly better social activity. |
| 9 | Byles et al 2004 | Home health assessments for older Australian veterans and war widows  Australia | RCT  3 years  1082 veterans or war widows receiving full entitlements from the Australian Department of Veterans’ Affairs, and aged ≥70 years. | At home  Conducted by: Nurses, social workers, psychologists,  physiotherapists, and/or occupational therapists.  Components: Use of hearing aids, vision, dental care and dentures,  vaccinations, prescribed and over-the-counter medications, hypertension management, diabetes management, smoking status and desire to quit, body mass index, problems with feet, problems with leaking urine, self-rated health, difficulty sleeping, use of community services, Australian Nutrition Screening Initiative Checklist, Medical Outcomes Study physical function scale (selected items to assess mobility), brief Mini-Mental State Examination, Duke Social Support Index, Modified Geriatric Depression Scale. | To improve health-related quality of life, reduce hospital and nursing home admissions,  and reduce mortality | Usual care without home-based health assessments by health professionals and telephone follow-up | Quality of life  Intervention group had higher quality of life than control-group participants in the Physical Component Summary score and Mental Component Summary score.  Mortality rate  -No difference between groups in mortality rate. | Healthcare utilisation  - No difference in the probability of hospital admissions.  - Intervention group had significantly more nursing home admissions than control group. |
| 10 | Chi et al 2006 | Primary care  Hong Kong | RCT  12 months  925 older Chinese adults aged ≥65 years who attend the elderly health centres of the department of Health, Hong Kong special Administrative Region. | At the elderly health centre  Conducted by: Trained interviewers.  Components: General functioning, cognitive function, social support,  physical functioning, physical illnesses, living environment, formal service utilisation, medication. | To improve Chinese older adults’ physical and psychological well-being. | Patients were assessed using the Minimum Data Set-Home Care but profiles were not generated, and were treated according to the doctor’s usual practice. | Functional status  - No difference between groups in ADLs and IADLs  - No difference between groups in stamina.  Mental health  - Intervention group had improvements in mood and behaviour.  Cognition  - No difference between groups in cognition.  Chronic conditions  - No difference between groups in pain symptoms and pressure ulcer.  - Intervention group had poorer bowel incontinence.  -No difference between groups in bladder incontinence.  Others  - No difference between groups in ability to communicate.  - No difference between groups in alcohol use and abuse. | Others  - No difference between groups in ability to communicate.  - No difference between groups in alcohol use and abuse. |
| 11 | Cohen et al 2002 | Outpatient clinic  [Only the outpatient intervention and control study participants in the study were reviewed]  United States | RCT  1 year  1388 persons aged ≥65 years who were hospitalised on a medical or surgical ward, had length of stay of at least two days, and frailty. | Outpatient geriatric evaluation and management  Conducted by: Geriatrician, social worker, and nurse.  Components: Medical history and physical examination, functional, cognitive, affective, and nutritional status, caregiver’s capabilities, patient’s social situation, and geriatric syndromes such as incontinence or falls. | To promote survival and functional status. | Usual care was providing at least one follow-up appointment at an appropriate outpatient clinic. | Functional status  - No difference between groups in BADL score, IADL score, and physical performance.  Quality of life  - No difference between groups in SF-36 scores for physical functioning, physical limitations, emotional limitations, and social activity, bodily pain.  - Intervention group had improved SF-36 scores for energy, general health, and mental health.  Mortality  No difference between groups for relative risk of death. |  |
| 12 | Drennan et al 2005 | Primary care  United Kingdom | Mixed methods  320 persons aged ≥75 years assessed to be high risk by general practitioners. | Primary care  Conducted by: Two persons from the 6 members of the multidisciplinary  health and social care team.  Components: 24 domains on health and social care. | To reduce unmet health and social care needs of high-risk elderly, reduce isolation, enable mainstream services to effectively focus delivery, and foster self-help for older people to promote quality of life. | NA (no controls) | Mental health  - The 15-item GDS was used on 247 participants, and the study found that 7% (n = 23) scored 6 and above, an indicator of clinical depression.  Cognition  - The Abbreviated Mental Test Score (AMTS) was used on 264 participants and 20% (n = 44) had a score of 7 or less, indicating problems with cognition.  Unmet needs  - 226 participants (71%) had no unmet needs. Most common unmet needs were problems with mobility. |  |
| 13 | Eckerbald et al 2016 | Ambulatory Geriatric Unit  Sweden | RCT  24 months  242 persons aged ≥75 years with  3 or more concomitant medical diagnoses and 3 or more hospitalisations during the preceding year. | At home  Conducted by: Trained registered nurses or a registered occupational therapist.  Components: Medical, psychological, functional. | To reduce the prevalence or burden of chronic disease symptoms. | Usual health and social care, including healthcare provided by the primary care, inpatient and outpatient hospital care and social care. | Chronic conditions  - Symptom trajectory of 31 chronic disease symptoms were assessed using the Memorial Symptom Assessment Scale (MSAS). There was no significant difference between groups for prevalence, burden or trajectory of symptoms. |  |
| 14 | Ekdahl et al 2015 | Ambulatory Geriatric Unit  Sweden | RCT  24 months  382 persons (208 intervention, 174 controls) aged ≥75 years who received inpatient hospital care 3 or more times in the previous 12 months and had 3 or more concomitant medical diagnoses. | At home  Conducted by: Registered nurse and registered occupational therapist.  Components: Hearing and vision problems, independence in ADLs, cognition, sense of security in care, health-related quality of life. | To reduce hospitalisation, increase sense of security in care interaction, and improve quality of life. | Usual health and social care (i.e., health care provided by primary care centers, inpatient and outpatient hospital care, and social care as usual), with access to ambulatory geriatric unit. | Quality of life  No difference in HR-QoL was observed.  Mortality  - The intervention group showed trends of reduced mortality rate.  . | Healthcare utilisation  - No difference between groups in no. of hospitalisations, but no. of inpatient days was lower in the intervention group.  Cost  Costs for the intervention and usual care were 33,371£ and 30,490£, which were not significantly different.  Patient satisfaction  - The intervention group had an increased sense of security in care interaction |
| 15 | Ekdahl et al 2016 | Ambulatory Geriatric Unit  Sweden | RCT  36 months  382 persons (208 intervention, 174 controls) aged ≥75 years who received inpatient hospital care 3 or more times in the previous 12 months and had 3 or more concomitant medical diagnoses. | At home and via phone  Conducted by: Registered nurse and registered occupational therapist.  Components: Hearing and vision problems, independence in ADLs, cognition, sense of security in care, health-related quality of life. | To improve clinical outcomes and reduce costs. | Usual social and health care, delivered at home, in primary care centers, and in the hospital. | Mortality  - The intervention group had a significantly lower mortality rate. | Healthcare utilisation  - No difference between groups in nursing home use.  - No difference between groups in mean number of hospitalisations.  - No difference between groups in mean number of inpatient days.  Cost  - No difference between groups in mean overall costs. |
| 16 | Faul et al 2009 | Home health care and self-management support for older adults with chronic conditions.  United States | Pre-post design  12 weeks  73 persons aged ≥65 years with chronic conditions and no ongoing home health care. | At home  Conducted by: Physical therapist, physical therapist student, social worker student.  Components: Cognition, functional status, physical mobility, mental health, physical home environment, chronic diseases, self-management, self-rated health. | To provide non-medical intervention strategies for the management of  chronic conditions. | Not applicable (no controls)  CGA + self-management of care plan (n=39)  CGA + telephone support for management of care plan (n=34)  No group with usual care without CGA. | Functional status  - Significantly improved functional status and physical mobility  Frailty and falls  - Significantly improved physical home environment in terms of reduced fall hazards.  Mental health  - Significantly improved mental health, adjusted for  hearing ability.  Health status  - Significantly improved self-rated health.  Chronic conditions  - Non-significant improvement in self-efficacy for chronic disease management. |  |
| 17 | Fenton et al 2006 | General practice  United States | Case control  20 months  583 persons (146 cases, 437 controls) aged ≥65 years who attended the 2 physician practices in the study and enrolled into the health plan from 2 years before their index visit with the geriatrician until either death or the end of the study. | Primary care practice  Conducted by: Fellowship-trained geriatrician.  Components: (1) standardized assessment of psychosocial, cognitive, and physical function and physical activity; (2) screening for pain, depression, dementia, urinary incontinence, fall risk, and substance abuse; (3) review for use of medications with frequent adverse side effects in elderly patients; and (4) focused physical  examination. | To reduce healthcare utilisation and costs. | Controls were from the population of patients over age 65 years with primary care physicians at 3 clinics that serve a community with sociodemographic characteristics similar to the community served by the intervention clinic. | Mortality  - No significant difference in mortality rate.  Medications  - No significant difference in rate of high-risk prescriptions. | Healthcare utilisation  - No significant difference in rates of specialty visits, outpatient visits, nursing home admission.  Cost  - Relative to matched controls during follow-up, total health care costs were 26.3% lower among intervention subjects |
| 18 | Fletcher et al 2004 | General practice  United Kingdom | RCT  3 years  8,797 persons aged ≥75 years from the general population. | At home  Conducted by: Nurse.  Components: Cognition, mental health, functional, physiological, social | To provide better care to older adults via universal or targeted assessment. | No usual care. All participants had either universal assessment under geriatric management, universal assessment under primary care, targeted assessment under geriatric management, or targeted assessment under primary care.  Universal = In-depth assessment Targeted = Only patients with ≥3 problems from the brief assessment underwent in-depth assessment. | Quality of life  - Geriatric management significantly improved mobility, social interaction and morale compared to primary care management.  Mortality  - No difference between groups in mortality rate. | Healthcare utilisation  - No difference among groups in institutional and hospital admissions.  Quality of care  - Universal assessment significantly improved home-care, compared to targeted assessment. |
| 19 | Godwin et al 2016 | Intensive care management program  Canada | RCT  12 months  143 persons aged ≥80 years functioning well cognitively and living independently in the community. | At home  Conducted by: Primary Care Nurse Specialist  Components: ADLs and IADL, symptomatology, medication usage, compliance and knowledge by medication review, safety issues, including risk of falls, use of stoves and other potentially dangerous appliance, general home and personal hygiene and maintenance, understanding of their medical/health conditions to determine their need for education, and need for community services. | To improve quality of life, symptoms, satisfaction with care and utilization of community and medical services, in independent community living elderly. | Usual ongoing care from their family doctors, and any other services available through government services that they chose to access. | Quality of life  - No significant difference in QoL measured using SF-36  and CASP-19.  Chronic disease  - No significant difference in symptomology using the  Comorbidity Symptom Scale. | Healthcare utilisation  - No significant difference in the use of formal, intermediate, or informal community services.  - No significant differences between in healthcare utilisation of family physician visits, emergency room visits, hospitalizations or use of diagnostic services.  Patient satisfaction  No significant difference in patient satisfaction using PSQ-18. |
| 20 | Hebert et al 2001 | Nurse-led multidimensional preventive programme  Canada | RCT  1 year  494 persons on the Quebec Home Insurance Plan aged ≥75 years | At home  Conducted by: Trained nurse.  Components: Medication, cognitive function, depression, balance or risk of falling, orthostatic hypotension, environmental risks, social support, nutrition, arterial hypertension, vision, hearing, incontinence. | To reduce functional decline of older people. | Usual health care without the nurse-led multidimensional preventative  program. | Functional status  - No difference in the Functional Autonomy Measurement System (SMAF).  - No difference in relative risk of functional decline.  Mental health  - No difference in Depuy's General Well-being Schedule (GWBS) on anxiety, depression, positive well-being, self-control, vitality, and general health. | Social support  - No difference in the Social Provisions Scale (SPS) on perceived social support. |
| 21 | Hoogendijk et al 2016 | Geriatric Care Model for frail older adults in primary care  Netherlands | RCT  24 months  1147 patients across 35 primary care practices, aged ≥65 years, and had a PRISMA-7 score of 3 or more. | At home  Conducted by: Practice care nurse  Components: Identification of care needs and health risks, including preventive health,  Cardio-respiratory conditions, health promotion, depression and anxiety, urinary incontinence, pain, social functioning, falls, tobacco and alcohol use, medication management. | To improve QoL in frail older adults. | Usual care was not restricted in any way. | Functional status  - No difference in ADL and IADL limitations.  Quality of life  - No difference in QoL measured using SF-12 and EQ-5D.  Mental health  - No difference in psychological well-being.  Health status  - No difference in self-rated health. | Healthcare utilisation  - No difference in total hospital admissions and acute hospital admissions  Social functioning  - No difference in social functioning |
| 22 | Imhof et al 2012 | In-Home Health  Consultation Program  Switzerland | RCT  9 months  413 persons aged ≥80 years who are german-speaking. | At home  Conducted by: Advanced practice nurse.  Components: Demographic variables, living situation, family network, and health status (mobility and falls, pain, vision and hearing ability, sleep pattern, bladder control, nutritional status, substance use, cognition, and use of medications and aides for mobility). Clinical tests were included for vision (Amsler-Gitter Test), gait, balance, and strength, tandem stand, timed five-chair-rise test, and screening for malnutrition (Mini Nutritional Assessment), and depression (Geriatric Depression Scale GDS-4). | To increase quality of life, reduce adverse health outcomes, and reduce healthcare utilisation. | Received healthcare services as usual provided by community health nurses and physicians, and covered by the participant's mandatory health insurance. | Quality of life  - No significant difference in QoL.  Frailty and falls  - The intervention group had a lower relative risk of falls and consequences of falls.  Acute adverse events  - Intervention group had a lower relative risk of acute events,  defined as acute health symptoms that required action, | Healthcare utilisation  - The intervention group had a lower relative risk of hospitalisations. |
| 23 | Kang et al 2020 | Primary care (medical centre and public health centre)  South Korea | Pre-post, without controls  Mean of 5.1 months  362 persons aged ≥65 years who regularly visited primary medical institutions at the regions where study was conducted. | Outpatient medical centre and public health centre  Conducted by: Trained nurses  Components: Comorbidity, physical function, cognitive function, quality of life, drugs, and nutrition. | To improve physical function, quality of life, medication, and nutrition. | Not applicable (no controls) | Functional status  - For physical function, mean time of the timed up and go test reduced significantly.  - Mean gait speed increased significantly.  - Mean grip strength of the female participants increased significantly. Mean grip strength of male participants did not change significantly.  Quality of life  - For health-related QoL, mean EQ-5D score for each domain decreased  Significantly, including mobility, self-care, usual activities, pain/discomfort, and anxiety/depression.  Polypharmacy  - Significant decrease in proportion of participants with polypharmacy.  Nutrition  - Significant decrease in proportion of participants at risk of malnutrition or were malnourished. | Patient experience  - Participants were very satisfied  with the CGA, with 282 (92.5 %) of evaluators being very satisfied, and 19 (6.2 %) evaluators were satisfied.  - Exercise training was  satisfactory, with 137 (44.9 %) evaluators indicating they were very satisfied, and 30 (9.8 %) indicating they were satisfied.  - Health lectures were satisfactory, with 193 (63.3 %) evaluators being very satisfied, and 46 (15.1 %) were satisfied.  - Overall evaluation of the model was positive, with an average score of 9.1 out of 10. |
| 24 | King et al 2018 | Primary care with gerontology nurse specialists  New Zealand | RCT  1 year before and after intervention  1400 persons aged ≥75 years enrolled in one of the primary healthcare practices that will implement the new care model. | At home  Conducted by: Specialist Gerontology Nurse.  Components: Body systems (respiratory, cardiac, neurological, gastrointestinal, musculoskeletal and bladder/bowel function), pain, medications, potential social issues, functional ability, cognitive impairment, depression. | To reduce healthcare utilisation in high needs older persons. | Usual care was provided through the primary healthcare practice, and did not involve screening for frailty or intensive care management. | Mortality rate  - No significant difference between groups in risk of mortality  . | Healthcare utilisation  - No difference in hospital admissions, emergency department visits, hospital readmissions, length of stay, or residential care admission.  - Relative risk of using community physiotherapy was significantly higher in the intervention group, compared to the control group |
| 25 | Li et al 2010 | Outpatient community hospital  Taiwan | RCT  6 months  310 persons aged ≥65 years living in neighbourhoods within 15min walking distance from the community hospital. | Community hospital on an outpatient basis  Conducted by: Nurses.  Components: Geriatric syndromes (falls, incontinence, polypharmacy, sleep disturbance, nutrition, pain); cognition; depression; nutrition; functional (visual acuity); physical; orthostatic hypotension screening | To improve frailty status in older adults. | Usual care and only receiving the frailty screening evaluation. | Functional status  - No difference between groups in the Barthel Index.  Frailty and falls  - No difference between groups in the likelihood to have a better outcome and likelihood to deteriorate in frailty status measured with the FFC. |  |
| 26 | Liimatta et al 2019 | Preventative home visits  Finland | RCT  2 years  422 persons aged ≥75 years not receiving home help or nursing services. | At home.  Conducted by: Nurse, physiotherapist, social worker.  Components: Functioning, Mental Capability, health status, health and social services present, mobility, strength, ADLs, IADLs, financial and other social service needs. | To prevent poor QoL and reduce mortality by preventative home visits. | Usual health and social care offered by the municipality. | Quality of life  - Intervention group had significantly improved QoL after 1 yr, but after the visits ended, there was no difference between groups at the 2^nd^ year.  Mortality  No difference between groups |  |
| 27 | Lin et al 2012 | Outpatient geriatric evaluation and management service  Taiwan | Pre-post design  12 months  Total of 140 persons aged ≥80 years with any health conditions, and aged ≥65 years with multiple complex care needs, or more than 3 co-morbid chronic diseases, or with geriatric syndrome. | Outpatient geriatric evaluation and management service in Taipei Veterans General, a tertiary medical centre.  Conducted by: Research nurses  Components: Physical Function,  IADLs, cognitive function, mood status, delirium, falls, incontinence  nutritional status, QoL, social care resource. | To provide geriatric evaluation and management services to older adults with any health condition, multimorbidity, or geriatric syndrome (immobility, instability, intellectual, impairment, incontinence, and iatrogenesis) | Not applicable (no controls) | Quality of life  - QoL was significantly improved.  - Among survivors of study period, QoL gained from the service model was estimated to be 4.1 QALY.  Polypharmacy  - Among survivors of the study period, number of long-term oral medications were significantly reduced. |  |
| 28 | Mazya et al 2019 | Outpatient care for older adults with multimorbidity and high healthcare utilisation  Sweden | RCT  24 months  360 persons aged ≥75 years with 3 or more chronic conditions 3 or more inpatient admissions the past 12 months. | At home and via phone  Conducted by: Nurse and social worker (home), pharmacist (phone).  Components: Medical, functional, psychological, cognitive, social. | To reduce frailty and mortality of older adults with multi-morbidity living at home. | Usual care was medical and social care, access to an acute care hospital, 10 primary care centres, and home care. | Mortality rate  - No difference between groups in mortality rate.  Frailty and falls  - Intervention group had significantly lower proportion of frail patients.  - Intervention group had a significantly higher proportion of pre-frail patients. |  |
| 29 | Monteserin et al 2010 | Primary care  Spain | RCT  18 months  620 persons aged ≥75 years who has access to primary care health centre. | Primary care health centre  Conducted by: Nurse.  Components: Socio-demographics, perceived health status, sensory  evaluation (sight and hearing), falls,  urinary incontinence, prescribed medications, comorbidity, functional status, IADL, neuropsychological status, cognitive status, nutritional status and social support. | To reverse risk of frailty and reduce morbidity and mortality for patients living in the community. | Usual care was assessment and care by general practitioners, without recommendations from nurse-led group sessions or recommendations from a geriatrician to a nurse or general practitioner. | Mortality  - No difference between groups for risk of mortality, among those at risk of frailty and not at risk of frailty.  Frailty and falls  - Intervention group had a significantly lower proportion of who went from not at risk for frailty to at risk of frailty.  - The intervention group had a significantly greater proportion who reserved their frailty status. | Healthcare utilisation  - Among those at risk of frailty, the intervention group had a significantly lower risk of being admitted to nursing homes.  - Among those not at risk of frailty, no difference between groups for being admitted to nursing homes. |
| 30 | Ploeg et al 2010 | Preventive primary care outreach.  Canada | RCT  12 months  719 persons aged ≥75 years at risk of functional decline. | At home  Conducted by: Nurse.  Components: Quality of Life, health status, costs of health and social services, functional status,  self-rated health. | To reduce risk of functional decline. | Not specified. | Functional status  - No significant difference between groups.  Quality of life  - No significant difference between groups for QALY.  Self-rated health  - No significant difference between groups. | Healthcare costs  - No significant difference between the two groups in the costs of prescription drugs, health and social services, or prescription drugs and health and social services combined. |
| 31 | Romskaug et al 2020 | Primary care practices  Norway | RCT  24 weeks  158 persons aged ≥70 years who used at least 7 systemic medications taken regularly,  and had their medications administered by the home nursing service. | Primary care practice  Conducted by: Physician trained in geriatric medicine, supervised by a senior consultant.  Components: Medical history, systematic screening for current problems, clinical examination of the patient, relevant supplementary test, and detailed review of each medication in use, with emphasis on indication, dosage, possible adverse effects, and interactions. | To Improve health-related quality of life in home-dwelling older patients that have polypharmacy. | Usual care was provided without the geriatric assessment, collaborative plan, and clinical follow up by the geriatrician or family physician. | Functional status  - No difference between groups for disability, as assessed with the Functional Independence Measure.  Quality of life  - No difference between groups in QoL.  Frailty and falls  - No difference between groups in number of falls.  Mortality rate  - No difference between groups in mortality rate.  Medication appropriateness  - Medication appropriateness as assessed by the Medication Appropriateness Index and the Assessment of Underutilization improved in the intervention group compared with the control group at 16 weeks and 24 weeks.  Others  - No differences between groups regarding orthostatic blood pressure, weight, relative stress, the number of days the patient spent in his or her own home during follow-up. | Healthcare utilisation  - No difference in the odds of being hospitalised for the intervention group, compared to the controls.  - No difference between groups for the use of the home nursing service, and admission to permanent institutional care. |
| 32 | Rubenstein et al 2007 | Primary care  United States | RCT  3 years  532 persons aged ≥65 years who had at least one clinic visit at the ambulatory centre in the previous 18 months and deemed high risk (impaired in 4 or more of 10 Geriatric Postal Screening Survey questions). | Over the phone and at a geriatric assessment clinic  Conducted by: Physician assistant case manager (phone), and geriatric medicine faculty, physician assistant, and internal medicine house staff (at clinic).  Components: Physical health, functional status, mental health, social and environmental status | To improve recognition of geriatric conditions and healthcare outcomes through a system of screening, assessment, referral and follow-up within primary care. | Usual care and serious conditions were followed up by providers. | Functional status  - No differences between groups in ADLs and IADLs.  Frailty and falls  - No significant difference between groups in prevalence or severity  of falls.  Chronic conditions  - No significant difference between groups in prevalence or severity of urinary incontinence.  Mental health  - No differences between groups in GDS scores.  Health status  - No differences between groups in self-rated health | Healthcare utilisation  - No difference between groups in hospital utilisation and number of hospital days.  Quality of care  - Intervention group  was more likely to have medical conditions documented in the medical record and to receive an evaluation for the conditions.  - Intervention group was more likely to be referred to specialised services related to their geriatric conditions. |
| 33 | Stuck et al 2000 | In-home visits for disability prevention  Switzerland | RCT  3 years  791 persons aged ≥75 years in the health insurance list of community-dwelling residents in three zip code areas of Bern, and categorised as high-risk and low-risk for nursing home admission. | At home  Conducted by: 3 certified registered nurses with an additional degree in public health nursing based on an 8-month postgraduate course (Nurse A, B, C)  Components: Medical history, physical examination, haematocrit and glucose levels in blood, hearing, vision, nutritional status, oral health, appropriateness of medication use, safety in the home, ease of access to external environment, social support. | To delay the onset of disabilities in older people. | Usual care without comprehensive geriatric assessments, individual recommendations, and in-home visits. | Functional status  - Among low-risk subjects, the intervention group were less dependent in IADL, compared to controls. Among high-risk subjects, there was no difference in IADL between intervention and controls.  - Subgroup analysis showed the among low-risk subjects visited by nurse A and nurse B, the intervention group had better IADL and ADL. | Healthcare utilisation  - Subgroup analysis showed the among low-risk subjects visited by nurse A and nurse B, the intervention group had fewer nursing home admissions  Cost  - Subgroup analysis showed the among low-risk subjects visited by nurse A and nurse B, the intervention group had net cost savings in the 3rd year (USD$1403 per person per year). |
| 34 | Suijker et al 2016 | Nurse-led home visits  Netherlands | RCT  24 months  2,283 persons aged ≥70 years with complex care needs. | At home  Conducted by: Community-care registered nurse.  Components: Somatic, psychological, functional, and social. | To function as a proactive intervention which addresses complex care needs and enable independent living in the community. | Usual care from a general practitioner. | Functional status  - No difference between group in Katz-ADL index scores.  Quality of life  - No difference between groups in health related-QoL, emotional wellbeing, and self-perceived QoL.  Mortality  - No difference between groups in all-cause mortality.  Frailty and falls  - No difference between groups in number of falls. | Healthcare utilisation  - No difference between groups for number of hospitalisations. |
| 35 | Suijker et al 2017 | Nurse-led home visits  Netherlands | RCT  12 months  2,283 persons aged ≥70 years with complex care needs. | At home  Conducted by: Community-care registered nurse.  Components: Somatic, psychological, functional, and social. | To function as a proactive intervention which addresses complex care needs and enable independent living in the community. | Usual care from a general practitioner. | Functional status  - No difference between group in Katz-ADL index scores.  Quality of life  - No difference between groups in QALY. | Cost/Cost effectiveness  - Cost of the intervention was significantly higher than usual care.  - To gain one point of improvement in modified Katz-ADL index, an additional EUR 21,884 needed to be invested in the intervention group.  - To gain one QALY, an additional EUR 287,879 needed to be invested in the intervention group. |
| 36 | van Hout et al 2010 | Nurse home visits  Netherlands | RCT  18 months  424 persons aged ≥75 years with frailty. | At home  Conducted by: Trained community nurse.  Components: Health risks and care needs using the Resident Assessment Instrument–Home Care version (RAI-HC). | To prevent adverse health trajectories in terms of poor functional status, disability in ADL and IADL, admissions into hospital, being placed in nursing homes or homes for the disabled, and mortality. | Usual care varied from no care at all, to regular primary care provider visits, to home care involvement. | Functional status  - No difference between groups in functional status, and disability in ADL and IADL.  Mortality rate  - No difference between groups in mortality rate. | Healthcare utilisation  - No difference between groups for hospital admittance, and placement into nursing homes or homes for disabled older persons.  - Among persons with the poorest self-rated health (EQ5D < 55), intervention group had a significantly higher risk to be admitted to a hospital compared with controls.  - A higher risk of acute hospital visits was found in the intervention group who had two or more chronic diseases |
| 37 | van Leeuwen et al 2015 | Chronic care model for frail older adults in primary care  Netherlands | RCT  24 months  1,147 frail older adults aged ≥65 years with PRISMA-7 scores of 3 or more. | At home  Conducted by: Registered nurses with experience in geriatric nursing.  Components: Health and care needs identified from the  web-based Community Health Assessment version 9.1 of the Resident Assessment Instrument. | To target health risks and care needs at an early stage, to stimulate active involvement of older adults in the care process, and to improve coordination between healthcare professionals. | Usual care in primary care i.e., older adults consult primary care physicians on their own initiative | Functional status  - No difference between groups in ADLs and IADLs.  Quality of life  - No difference between groups in using SF-12 physical and mental scales. | Cost effectiveness  For  SF-12 the probability of the intervention  being cost-effective was 0.76 if decision-makers are willing  to pay $30,000 per point improvement on the SF-12 scales  (range 0–100). For other outcomes, probability of the intervention being cost-effective was low |

**ADL:** Activities of Daily Living; **BI:** Barthel Index; **CGA:** Comprehensive Geriatric Assessment; **FFC:** Fried Frailty Criteria; **GDS:** Geriatric Depression Scale; **IADL:** Instrumental Activities of Daily Living; **PRISMA:** Program of Research to Integrate the Services for the Maintenance of Autonomy; **QoL:** Quality of Life
